# ﻿*Pullimosina* (*Pullimosina*) *turfosa* sp. nov. and other Sphaeroceridae (Diptera) from peat bogs in the North Caucasus (Russia)

**DOI:** 10.3897/zookeys.1132.94579

**Published:** 2022-11-25

**Authors:** Jindřich Roháček, Andrey A. Przhiboro

**Affiliations:** 1 Silesian Museum, Nádražní okruh 31, CZ-746 01 Opava, Czech Republic Silesian Museum Opava Czech Republic; 2 Zoological Institute, Russian Academy of Sciences, St Petersburg, 199034 Russia Zoological Institute, Russian Academy of Sciences St Petersburg Russia

**Keywords:** biology, brachyptery, distribution, lesser dung flies, Limosininae, relationships, taxonomy, tyrphophily

## Abstract

The first data about Sphaeroceridae occurring on eight montane and foothill peat bogs of various types in the North Caucasus (Russia) are presented. A total of 38 species has been recorded and their affinity to peat-bog habitats is discussed. A single species is classified as a tyrphobiont, viz. the strongly brachypterous Pullimosina (Pullimosina) turfosa**sp. nov.** being strictly associated with *Sphagnum* hummocks in peat bogs. This new species is described and illustrated in detail and its relationships, biology, and wing reduction are discussed. Only three species are considered tyrphophilous or probably tyrphophilous, viz. *Ischioleptanitida* (Duda, 1920), Phthitia (Kimosina) longisetosa (Dahl, 1909), and *Spelobiaibrida* Roháček, 1983. The majority of recorded species do not have close affinity to peat bogs and are treated as tyrphoneutral, and *Rachispodahostica* (Villeneuve, 1917) is probably tyrphoxenous due to occasional occurrence in a peat bog. Species composition of Sphaeroceridae on Caucasian peat bogs is discussed in comparison to those known from peat bogs in other parts of Europe. Taxonomic notes are given on Minilimosina (Svarciella) species of the *M.vitripennis* group. Six species (including *P.turfosa***sp. nov.**) are new additions to the fauna of Russia.

## ﻿Introduction

Sphaeroceridae or Lesser dung flies are a relatively large family of Acalyptratae, with more than 1,800 described (and at least 5,000 estimated) species in the world fauna of Diptera (Papp and Roháček 2021). The family is richly represented in all biogeographic regions except for extreme Arctic and Antarctic areas. The European fauna of Sphaeroceridae is diverse, with approximately 260 named and more than 270 presupposed species (Pape et al. 2015). Probably all species are saprophagous (more precisely, microsaprophagous) because both larvae and adults are feeding on liquids with microorganisms and decomposed organic substances from decaying animal (excrement, carrion), vegetal (dead plants and their remnants including forest litter), and fungal (sporocarps of macrofungi) organic matter (Richards 1930; Roháček 1998; Papp and Roháček 2021). This trophic strategy enables these flies to colonize very diverse habitats, including nutrient-poor (oligotrophic) and strongly acidic peat bogs.

Insect fauna of mires (peat bogs in a broad sense, i.e., peatlands where peat is currently being formed and accumulating) has been relatively well studied both in Europe and North America (for reviews, see Marshall et al. 1999; Keiper et al. 2002; Spitzer and Danks 2006; Batzer et al. 2016) but most of the published faunal and ecological studies are devoted to Lepidoptera and Coleoptera while even in most complex studies (e.g., Harnisch 1925; Peus 1928, 1932; Rabeler 1931; Pax 1937; Krogerus 1960; Nelson 1971; Coulson and Butterfield 1985; Drake et al. 1989; Boyce 2004; Spungis 2008; Sushko 2012; Anderson et al. 2017), as a rule, Diptera are treated only marginally, with faunal studies mostly devoted to selected families of this order (e.g., Rief 1996; Salmela 2004; Przhiboro and Paasivirta 2012). This is particularly true for Sphaeroceridae: the reliable data on the occurrence of sphaerocerid species on peat bogs are not very numerous and can mostly be found in studies published by specialists on this group, viz. Roháček (1984), Roháček and Barták (1999) (in the Czech Republic), Kuznetsova (1987) (in Latvia), Pitkin et al. (1985) and Holmes et al. (1992) (in Great Britain), and Marshall (1994, 1997) (in Canada). Additional data on Sphaeroceridae from peat bogs can be found in publications by other dipterists, e.g., Elberg (1969, 1971), Taillefer and Wheeler (2010, 2011), and Przhiboro (2012).

This study is devoted to Sphaeroceridae obtained from montane and submontane peat bogs in the North Caucasus by the second author. These mires are rare, mostly small and isolated habitats situated near the southernmost limit of the occurrence of this habitat type in Europe and the whole of the Palaearctic Region. They are characterized by specific environmental conditions and distinctive composition of vegetation (Botch and Masing 1979, 1983) with relict taxa of originally Boreal flora. The knowledge of insects in these peat bogs is poor and the composition of the fauna of Diptera remains practically unknown apart from small recent contributions dealing with Tephritidae (Evstigneev and Przhiboro 2021; Evstigneev and Glukhova 2022) and Anthomyzidae (Roháček and Przhiboro 2022).

## ﻿Materials and methods

### ﻿Material

A total of 119 adults of Sphaeroceridae was collected in peat bogs together with other macroinvertebrates in the scope of faunal and ecological studies of these mires in early May 2016, late May to early June 2018, and in September 2018. Most of the examined material is deposited in Zoological Institute, Russian Academy of Sciences, St Petersburg, Russia**(ZISP)**. Some duplicates are retained in Slezské zemské muzeum, Opava, Czech Republic**(SMOC**). Most specimens are preserved in 80% ethanol, only the type specimens and a few other voucher specimens (indicated in the list of material as “dry”) have been dried and mounted on pinned triangular cards to be deposited in dry collections of Diptera.

### ﻿Collecting methods

On each mire, most sampling effort was focused on collecting macroinvertebrates from dry and wet habitats where *Sphagnum* mosses dominate or are abundant. The following main sampling techniques were used in each mire: sweep-netting with aerial net over grassy vegetation (in different daytime periods and weather), yellow pan traps, pitfall traps, sifting substrate of *Sphagnum* cushions or hummocks located in drier places, trampling *Sphagnum* cushions located in wet places (like shorelines of in-mire lakelets), sweep-netting with aquatic net in puddles and along shorelines of lakelets, and sampling the substrate (*Sphagnum*, other plants, turf, and litter). The latter samples were washed in sieves (the smallest 0.25 mm mesh), then macroinvertebrates were extracted by flotation in a strong solution of NaCl combined with hand-sorting of the coarse fraction. However, most Sphaeroceridae were collected by sweep-netting; only a few specimens were obtained by sifting, yellow pan traps, and from the samples of substrata (indicated below as “sample no.”).

### ﻿Methods of preparation and study of postabdominal structures

Abdomens of some specimens were detached, cleared by boiling for several minutes in 10% solution of potassium hydroxide (KOH) in water, then neutralized in 10% solution of acetic acid (CH_3_COOH) in water, washed in water and subsequently transferred to glycerin. Postabdominal structures were dissected and examined in a drop of glycerin under binocular microscopes (Reichert, Olympus). Detailed examinations of genital structures were performed with a compound microscope (Zeiss Jenaval). After examination, all dissected parts were put into small plastic tubes containing glycerin, sealed with hot forceps and pinned below the respective specimens. Specimens with abdomen removed and terminalia dissected are indicated in the list of material by the abbreviation “genit. prep.”

### ﻿Drawing techniques and photography

Legs and details of the male and female genitalia were drawn by means of an Abbe’s drawing apparatus on a compound microscope (Zeiss Jenaval) at magnification 130–500×. Wings were photographed on an Olympus BX51 compound microscope with an attached digital camera (Canon EOS 1200D). Whole adult (dry-mounted) specimens and heads were photographed by means of a Canon EOS 5D Mark III digital camera with a Nikon CFI Plan 10×/0.25NA 10.5mm WD objective attached to a Canon EF 70–200mm f/4L USM zoom lens. The specimen photographed by means of the latter equipment was repositioned upwards between each exposure using a Cognisys StackShot Macro Rail and the final photograph was compiled from multiple layers (~ 40) using Helicon Focus Pro 7.0.2. The final images were edited in Adobe Photoshop CS6. All morphological illustrations were prepared by the first author.

### ﻿Measurements

Five characteristics of the new species were measured: body length (measured from anterior margin of head to end of cercus, thus excluding the antenna), index t_2_: mt_2_ (= ratio of length of mid tibia: length of mid basitarsus), wing length (from wing base to wing tip), wing width (maximum width), Cs_1_: Cs_2_ (= ratio of length of 1^st^ costal sector: length of 2^nd^ costal sector). All type specimens were measured.

### ﻿Presentation of faunistic data

Label data of the type specimens are presented strictly verbatim including information on form and color of all associated labels. Locality data of other specimens examined are given in brief form because all other information is given under the descriptions of localities. Localities are listed in the same order as in their descriptions below. Biological information obtained from the material examined and literature are given in the Comments paragraph. General distribution of species recorded are based on Roháček et al. (2001) and Marshall et al. (2011) unless mentioned otherwise. Species recorded from Russia for the first time are marked by * preceding the species’ name.

### ﻿Assessment of the affinity of species to mire habitats

The affinity of a species to peat-bog habitat has been judged by the first author based on his knowledge of the biology, autecology and distribution of the species. Four categories are differentiated according to the degree of association with bog habitats following Peus (1928, 1932), Roháček and Máca (1982), and Spitzer and Danks (2006):

1. tyrphobiont (**TB**) ‒ species strictly associated with peat-bog habitats (occur only in bogs),
2. tyrphophilous (**TPH**) ‒ species preferably associated with peat-bog habitats (characteristic of bogs but not confined to them),
3. tyrphoneutral (**TN**) ‒ species with a wide habitat tolerance (resident in bogs but also, often more successfully, in other habitats),
4. tyrphoxenous (**TX**) ‒ species coincidentally encountered in peat-bog habitats (non-resident vagrants which cannot survive in bogs).

These categories can be compared with those more generally used in North Europe (e.g., by Krogerus 1960) as follows: tyrphobiont = eucoenic (euzön), tyrphophilous = tychocoenic (tychozön), tyrphoneutral = acoenic (azön), tyrphoxenous = xenocoenic (xenozön).

### ﻿Morphological terminology

Morphological terminology follows that used for Sphaeroceridae by Roháček (1998) in the Manual of Palaearctic Diptera including terms of the male hypopygium. The “hinge” hypothesis of the origin of the eremoneuran hypopygium, re-discovered and documented by Zatwarnicki (1996), has been accepted and, therefore, the following synonymous terms of the male genitalia (emanating from other hypotheses) need to be listed (terms used first): ejacapodeme = ejaculatory apodeme, epandrium = periandrium, medandrium = intraperiandrial sclerite, phallapodeme = aedeagal apodeme. Morphological terms of the male postabdomen and genitalia are depicted in Figs 23–30, those of the female postabdomen in Figs 33–39. Abbreviations of morphological terms used in text and illustrations are listed below.

### ﻿Abbreviations of morphological terms used in text and/or figures

**A_1_** anal vein

**ac** acrostichal (seta)

**ads** additional (setulae) on frons

**asc** additional sclerite

**C** costa

**ce** cercus

**Cs_1_, Cs_2_** 1^st^, 2^nd^ costal sector

**CuA_1_** cubitus

**cx_1_** fore coxa

**dc** dorsocentral (seta)

**dp** distiphallus

**ea** ejacapodeme

**ep** epandrium

**f_1_, f_2_, f_3_** fore, mid, hind femur

**g** genal (seta)

**gs** gonostylus

**h** humeral cross-vein

**hu** humeral (= post-pronotal) (seta)

**hy** hypandrium

**ifr** interfrontal (seta)

**M** media

**ma** medandrium

**mt_2_, mt_3_** mid, hind basitarsus

**oc** ocellar (seta)

**occe** outer occipital (seta)

**occi** inner occipital (seta)

**ors** fronto-orbital (seta)

**pg** postgonite

**pha** phallapodeme

**pp** phallophore

**prg** pregonite

**pvt** postvertical (seta)

**R_1_** 1^st^ branch of radius

**R_2+3_** 2^nd^ branch of radius

**R_4+5_** 3^rd^ branch of radius

**S1–S10** abdominal sterna

**sc** scutellar (seta)

**stpl** sternopleural (= kat-episternal) (seta)

**T1–T10** abdominal terga

**t_1_, t_2_, t_3_** fore, mid, hind tibia

**va** ventroapical seta on t_2_

**vi** vibrissa

**vte** outer vertical (seta)

**vti** inner vertical (seta)

### ﻿Localities studied

For mire types and characteristics, we mostly follow the terminology and definitions adopted by Joosten et al. (2017). In general, they are in agreement with the terms used in recent entomological reviews on mires (Vitt 1994; Marshall et al. 1999; Keiper et al. 2002; Spitzer and Danks 2006, etc.).

**Figure 1.**
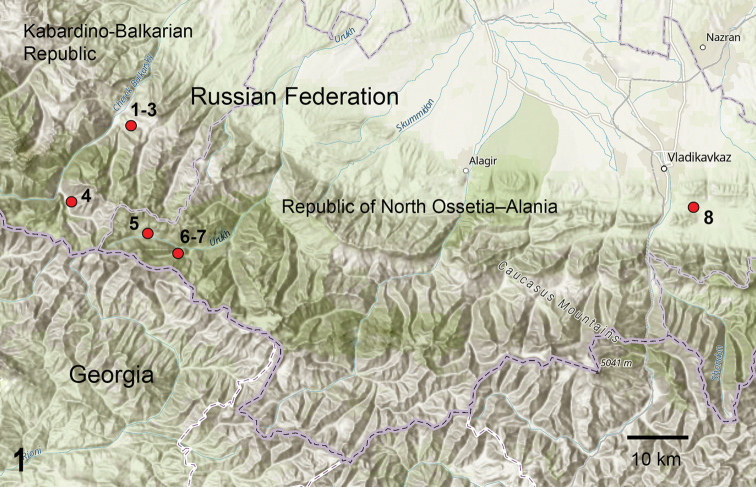
Map of the North Caucasus with position of mires under study. Localities on map; **1** Kurnoyatsu-1; **2** Kurnoyatsu-2; **3** Kurnoyatsu-3; **4** Ushtulu; **5** Chifandzar; **6** Kubus-larger; **7** Kubus-smaller; **8** Tarskoe. Map source: ArcGIS Online.

Eight montane and submontane mires (peat bogs) were studied, all situated in the central part of the northern slope of the Greater Caucasus Range (Fig. 1). Four mires (Fig. 1: localities 1–4) are located in Cherekskiy District of the Kabardino-Balkarian Republic (area of eastern Balkaria). The other four mires (Fig. 1: localities 5–8) are situated in the Republic of North Ossetia-Alania: Chifandzar and two bogs at Kubus Mountain in Irafskiy District (historical area of Western Digoria), and Tarskoe peatland in Prigorodnyy District (in Tarskaya Hollow).

**Figures 2, 3.**
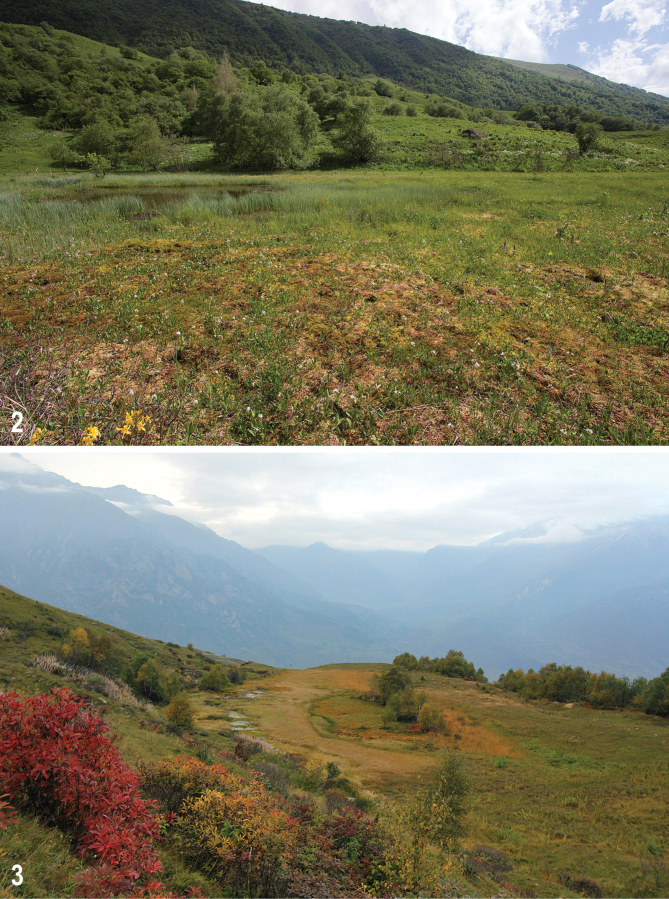
Mires under study **2** Kurnoyatsu-1, 6 June 2018 **3** Kurnoyatsu-2, 22 September 2018.

**Figures 4, 5.**
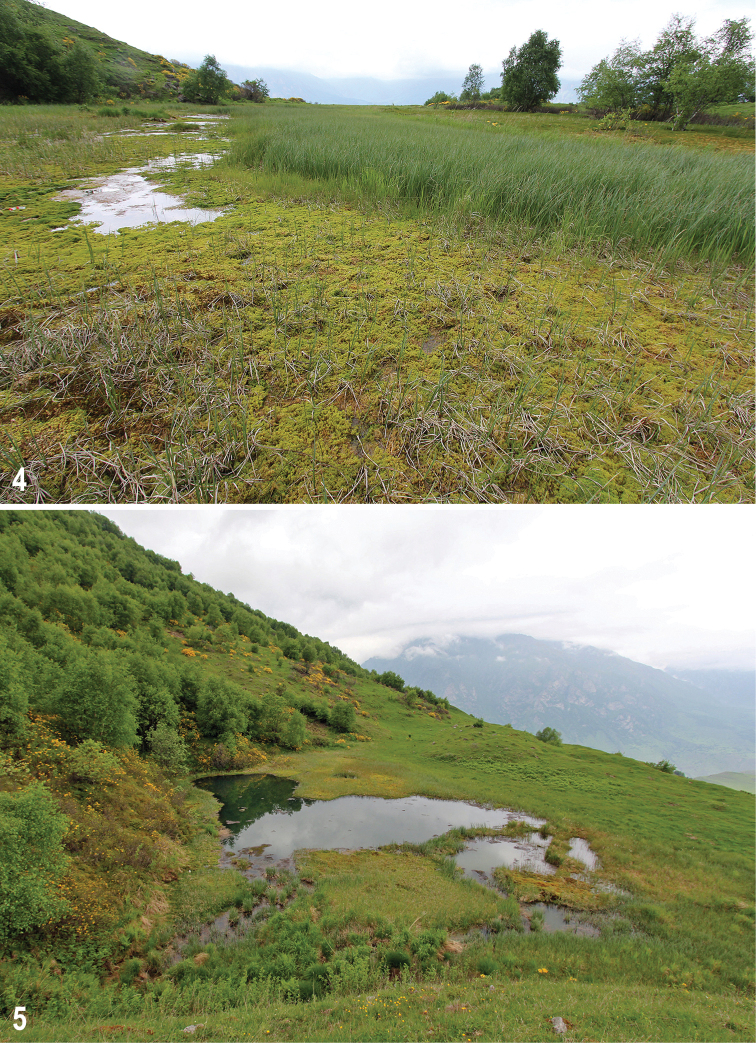
Mires under study **4** Kurnoyatsu-2, 7 June 2018 (close-up view) **5** Kurnoyatsu-3, 7 June 2018.

**Figures 6, 7.**
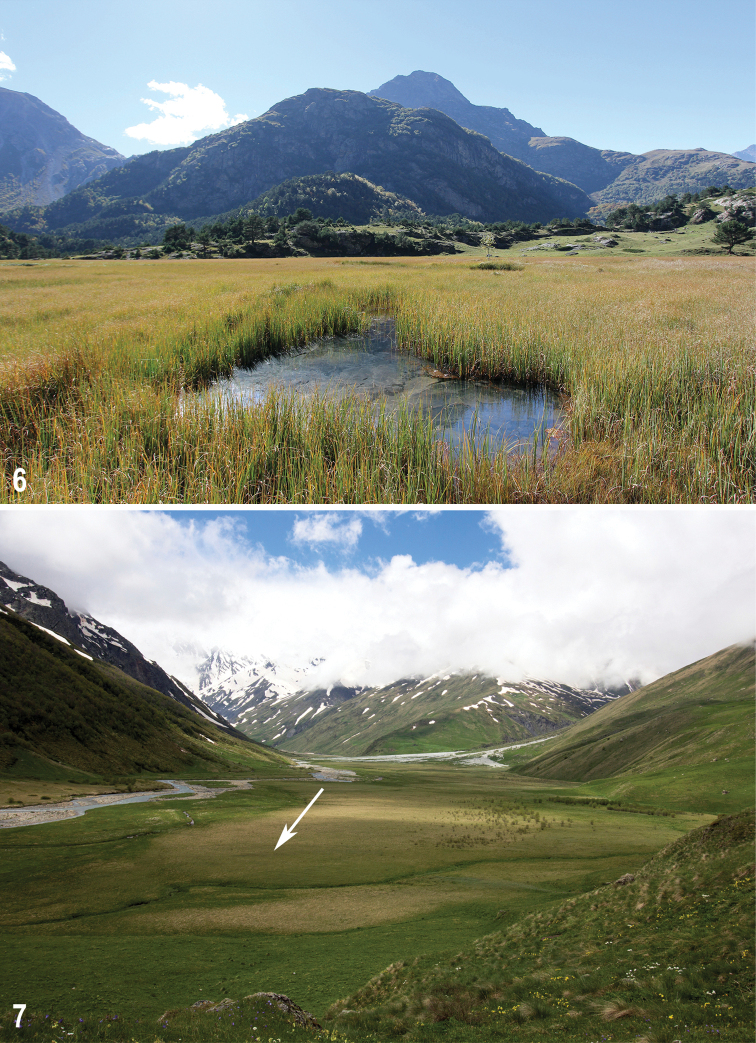
Mires under study **6** Ushtulu, 21 September 2018 **7** Chifandzar, 2 June 2018 (arrow indicates an area in which large *Sphagnum* hummocks are located).

**Figures 8, 9.**
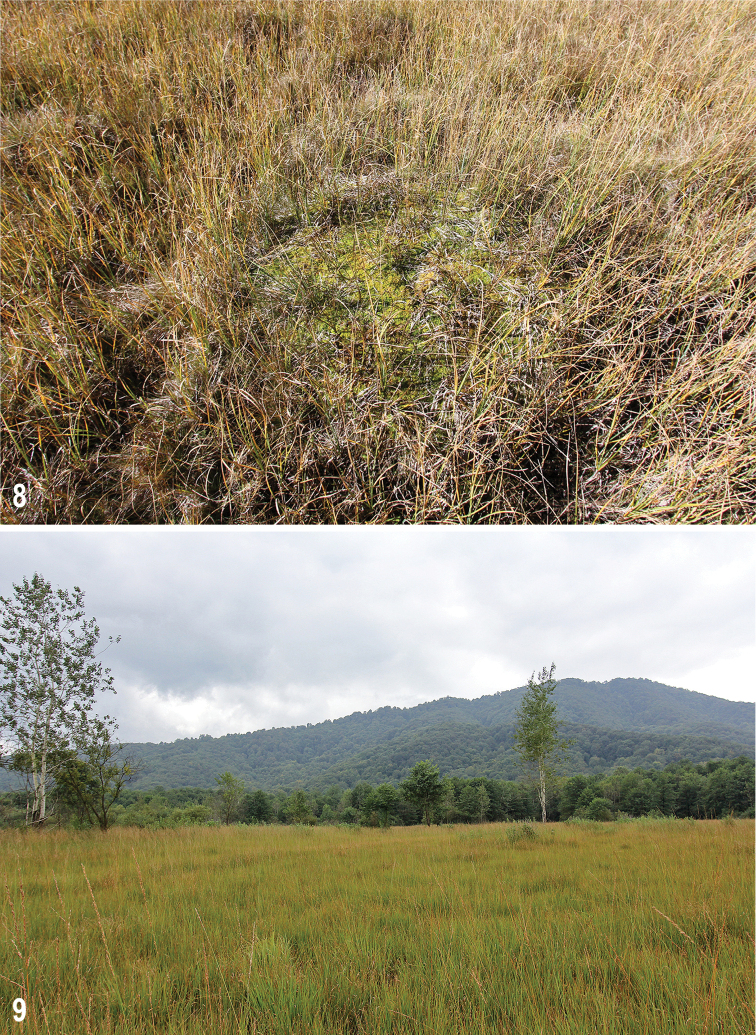
Mires under study **8** Chifandzar, 18 September 2018, a large *Sphagnum* hummock **9** Tarskoe, 11 September 2018.

**Figures 10, 11.**
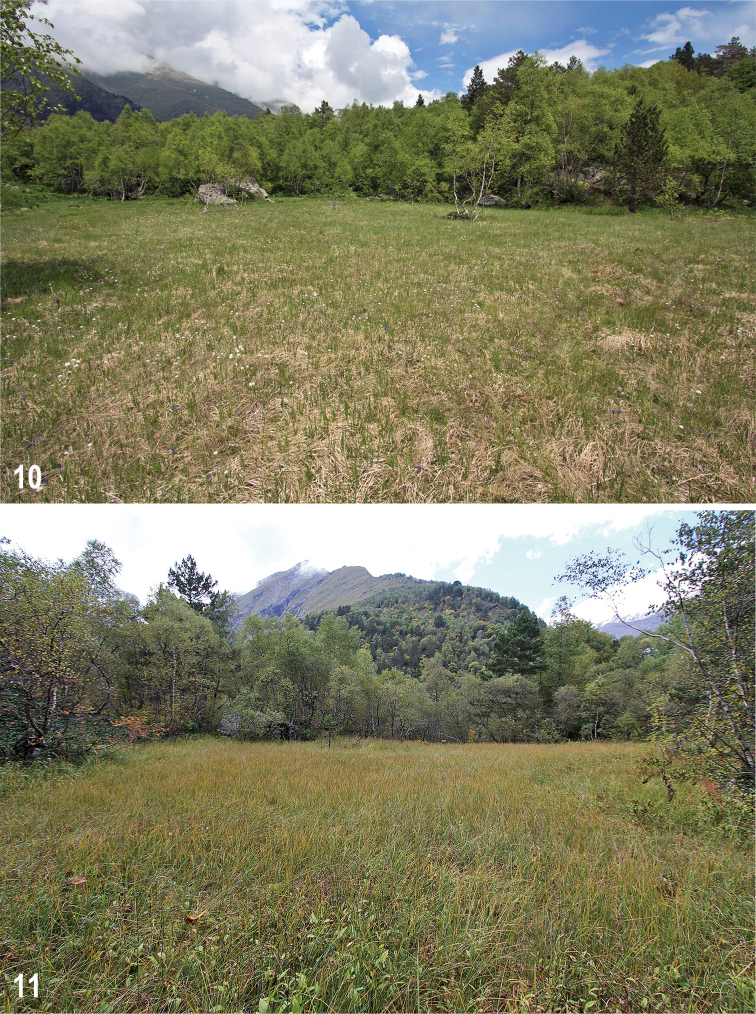
Mires under study **10** Kubus-larger, 4 June 2018 **11** Kubus-smaller, 12 September 2018.

The mires under study strongly differ in the size (0.0004 to ca. 0.5 km^2^), altitude (800 to 2290 m), trophic status (oligo- to eutrophic), origin, and type (*Sphagnum*-, *Carex*-*Sphagnum*- and *Carex*-dominated). Conditions of the study mires and previous publications about these mires were briefly reviewed in Prokin et al. (2019) and Prokina and Philippov (2019). The mires are briefly described below, with additional information given in Table 1. Photos of the mires under study (taken by the second author) are given as Figs 2–11. It should be said that in general the mires under study are ranging from a transitional mire to a rich fen; typical ombrotrophic bogs (i.e., fed solely on precipitation; raised or flat) are absent. Several mires are transient (mixed), i.e., include parts or sites which correspond to different hydromorphic and trophic types. All mires are unforested and flat.

### ﻿Mires of the Kabardino-Balkarian Republic

Three small transitional mires called here Kurnoyatsu-1, Kurnoyatsu-2, and Kurno­yatsu-3 (having no official names) are situated on the slope at left bank of the Kurno­yatsu River, ca. 3 km SE of Verkhnyaya Balkaria Village. These mires are situated in the alpine zone near the forest edge, on small flat terraces at different heights, 200–500 m from each other. The mires are limnogenous; each has a lakelet in the middle, with swing moor at shoreline. Relief of hummocks is not developed. Kurnoyatsu-1 (Fig. 2) and Kurnoyatsu-2 (Figs 3, 4) are transient (mixed) mires, with the fen changing into a bog: they have a large, dry, mostly *Sphagnum*-dominated, bog area (apparently ombrotrophic in the second case) and a wet *Sphagnum*- and/or *Carex*-dominated fen area around the lakelet. In Kurno­yatsu-3 (Fig. 5), dry habitat is not developed.

**Table 1.** Characteristics of study mires.

Region (area within)	Bog name	Coordinates	Altitude (m)	Mire area (m^2^)*	Trophic status	Habitats	Dominating species of *Sphagnum*	Dominating species of phanerogams
Kabardino-Balkaria (eastern Balkaria)	Kurnoyatsu-1	43.10062°N, 43.48418°E	1776	2600	oligotrophic to meso-oligotrophic	dry	*S.magellanicum*-coll. (*S.divinum*)	*Moliniacaerulea*, *Menyanthestrifoliata*
						wet (near lake)	*S.squarrosum*, *S.teres*, *S.flexuosum*, *S.obtusum*	*Carexrostrata*, *C.canescens*, C.?diandra, *Menyanthestrifoliata*
	Kurnoyatsu-2	43.09834°N, 43.47776°E	1810	4000	oligotrophic to meso-oligotrophic	dry	*S.fuscum*	*Rhododendronluteum*, *Empetrumnigrum*, *Carexrostrata*
						wet (near lake)	*S.fallax*, *S.flexuosum*	*Carexrostrata*, *C.canescens*, *Calamagrostis* sp.
	Kurnoyatsu-3	43.09714°N, 43.47950°E	1836	1500	oligotrophic to meso-oligotrophic	mostly wet	*S.fallax*, *S.flexuosum*	*Carexrostrata*, *Eriophorumangustifolium*
	Ushtulu	42.97457°N, 43.33263°E	1995	180000	eutrophic	dry	*S.warnstorfii*	*Carexrostrata*
						wet	*S.warnstorfii*	*Carexrostrata*
North Ossetia-Alania (western Digoria)	Chifandzar	42.91867°N, 43.51493°E	2289	520000	eutrophic	dry (large hummocks)	*S.teres*	*Carexrostrata*, *C.* sp., *Nardusstricta*
						wet	*S.subsecundum*	*Carexrostrata*, *Nardusstricta*
	Kubus-larger	42.89350°N, 43.57733°E	2077	2300	oligotrophic	dry	*S.capillifolium*	*Moliniacaerulea*, *Eriophorumangustifolium*, *Nardusstricta*
	Kubus-smaller	42.89350°N, 43.57733°E	2080	400	oligotrophic	mostly wet	*S.subsecundum*	*Carexrostrata*, *C.magellanica*, *C.lasiocarpa*, *Nardusstricta*
North Ossetia-Alania (Tarskaya Hollow)	Tarskoe	42.96311°N, 44.72636°E	800	62000	meso-oligotrophic	dry	*S.magellanicum*-coll. (*S.divinum*), *S.centrale*	*Moliniacaerulea*
						wet (near ditches)	*S.subsecundum*	*Moliniacaerulea*, *Carex* sp.

* Calculated from satellite images in SAS.Planet software (Bing-Satellite, ArcGIS.Clarity, Google-Satellite, Yandex-Satellite) using the photos of study bogs taken in 2016–2018. Some values given for the same bogs in Prokin et al. (2019) are erroneous.

Ushtulu mire (also called “Narzannoe”; Fig. 6) is situated in the valley of the Ka­rasu River (right tributary of the Balkarskiy Cherek River in its upper reach), at its right bank, 17 km SW of Verkhnyaya Balkaria Village, above the timberline. It is a 700-m long eutrophic, mostly high *Carex*-dominated (partially *Carex*-*Sphagnum*-dominated) rich fen of spring origin, with emissions of mineral groundwater (mostly spring-fed), without distinct relief of hummocks. There are secondary in-mire lakes surrounded by wide wet areas including swing moor at their shorelines, and drier areas outside.

### ﻿Mires of the Republic of North Ossetia-Alania

Chifandzar mire (Fig. 8) is situated in the valley of the Kharesidon River (upper reach of the Urukh River) and occupies a wide and flat fluvial terrace at the left bank of the river. It is the highest and largest (ca. 2 km long) mire in the study area, situated in the alpine zone above the timberline. Chifandzar is a *Carex*-dominated poor fen; most of its area is wet, with small brooks and a well-developed relief consisting of small hummocks and moist interspaces between them. Most of the mire area is almost lacking *Sphagnum*. Dry habitat (Fig. 9) is represented by ~ 15 larger hummocks (with *Sphagnumteres* predominant) located in the eastern part of the mire.

Two small transitional mires of Kubus, Kubus-larger (Fig. 10) and Kubus-smaller (Fig. 11), are situated on a flat saddle at the Kubus Mountain, 1 km W of its top. These mires are less than 100 m from each other, each surrounded by mixed forest. The larger bog has also the name “Tana glade”. It is distinctly drier, while the smaller bog is wet. Both are *Sphagnum*-dominated and rather uniform, without distinct hummocks.

Tarskoe peatland (Fig. 9) is located in Tarskaya Hollow, ca. 2.5 km W of Tarskoe Village. It is a foothill transitional mire, the lowest, situated ca. 100 km E of the other studied mires. As distinct from the other mires, Tarskoe is strongly modified by drainage and peat extraction which started in 1939; the peatland is crossed by numerous artificial ditches. Grass mesophilic assemblages dominate over most of the peatland area, which has a well-developed relief consisting of hummocks. *Carex*-*Sphagnum*-dominated habitats are confined mostly to ditches and also occur as a few patches in drier areas beyond ditches.

Three Kurnoyatsu mires are occasionally used for grazing horses, while Chifandzar and Tarskoe mires, for grazing cattle. Chifandzar and Kubus mires are located within the Alania National Park; Tarskoe peatland has a formal protection status as a regional natural monument; Ushtulu mire is situated within the borders of the Kabardino-Balkaria State High-Mountain Reserve; Kurnoyatsu mires have no protection status.

## ﻿Results

### ﻿ Pullimosina (Pullimosina) turfosa sp. nov.

Taxon classificationAnimaliaDipteraSphaeroceridae

3A4F67A7-17BD-5ACE-B47D-4E7C567A01EC

https://zoobank.org/3D55B25A-F2A7-4FAF-ACC2-D189349E7AFC

Figs 12–13 , 14–17 , 18–22 , 23–26 , 27–30 , 31–32 , 33–39

#### Type material.

**Figures 12, 13.**
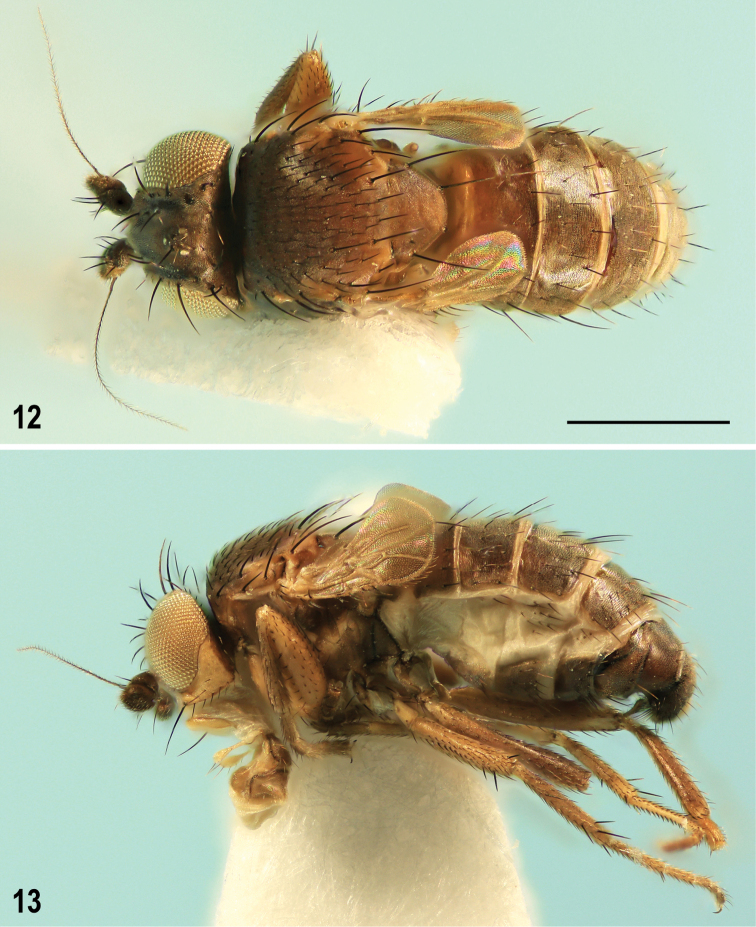
Pullimosina (Pullimosina) turfosa sp. nov. (male holotype) **12** whole body, dorsally **13** ditto, laterally. Scale bar: 0.5 mm.

***Holotype*** ♂ labelled: “Russia: N Ossetia, W Digoria, Chifandzar mire in Kharesidon River valley, 42.91867°N, 43.51493°E, 2289 m, sifting from *Sphagnumteres* hummocks, 18.ix.2018, A. Przhiboro leg.“, ”Holotypus ♂ Pullimosina (Pullimosina) turfosa sp. n., J. Roháček det. 2022“ (red label). The specimen is dried from ethanol and mounted on pinned triangular card, intact (deposited in ZISP, Figs 12, 13). ***Paratypes***: 6♂ 5♀ with same locality labels but with ”Paratypus [♂ or ♀], Pullimosina (Pullimosina) turfosa sp. n., J. Roháček det. 2022“ yellow labels; 3♂ 1♀ paratypes preserved in pinned microvial in glycerin, with abdomen detached, and terminalia dissected; others dry-mounted from ethanol and pinned as is the holotype; 1♂ 1♀ with wing removed for photography and also preserved in glycerin in pinned plastic tube below the specimen (4♂ 3♀ in ZISP, 2♂ 2♀ in SMOC). Other paratypes: 1♀, same locality data, but with “Sample Ч 14 (*Sphagnumteres*), 17.ix.2018“; 1♀, same locality data, but with “Sample Ч 9 (*Sphagnumsubsecundum*), 17.ix.2018“, both A. Przhiboro leg. (ZISP).

#### Etymology.

This strongly brachypterous species is named *turfosa* (= peaty, Latin adjective) owing to its strict association with *Sphagnum* hummocks in the type locality.

#### Description.

**Male** (Figs 12, 13). Total body length 1.27–1.64 (holotype 1.64) mm; general color brown to dark brown with greyish brown microtomentum, subshining dorsally (thorax, abdomen) and ventrally (abdomen), dull on thoracic pleuron.

**Figures 14–17.**
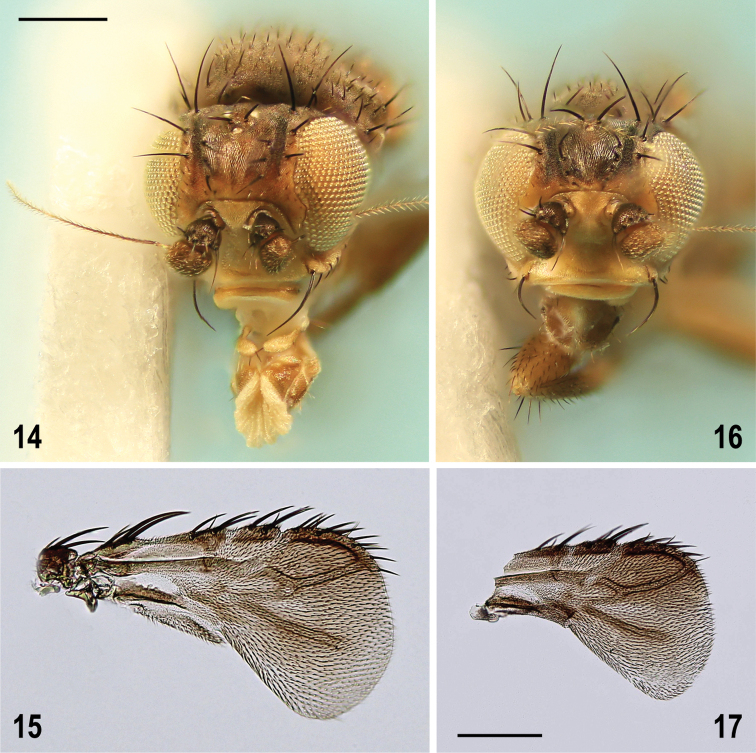
Pullimosina (Pullimosina) turfosa sp. nov. (holotype, paratypes) heads and wings **14** male head, frontally (holotype) **15** male right wing, dorsally (paratype) **16** female head, frontally (paratype) **17** female right wing, dorsally (paratype). Scale bars: 0.2 mm (**14, 16**); 0.1 mm (**15, 17**).

***Head*** (Figs 13, 14) higher than long (ca. 5:4), bicolorous, dorsally and posteriorly brown to pale brown, anteriorly and ventrally yellow to pale ochreous. Frons brown to pale brown, with anterior margin and orbits pale ochreous (Figs 14, 16), sparsely microtomentose and partly (mainly medially) shining; occiput dark brown with brownish grey microtomentum. Orbits, interfrontalia and ocellar triangle with paler greyish microtomentum; orbit separated from interfrontalia by dark brown dull stripe (shortened anteriorly, never reaching anterior margin of frons); frontal triangle indistinctly delimited but long, almost reaching anterior margin of frons, finely longitudinally microsculptured (Fig. 14), and more shining than rest of frons. Cephalic chaetotaxy (cf. Figs 12–14, 16): pvt present but reduced, hair-like but convergent and with apices almost meeting medially; occe and occi subequal (or occi slightly longer) and ca. two-thirds to three-fourths length of vti; vti normally thickest and longest of frontal setae; vte and oc only slightly shorter than vti; 2 ors, posterior almost as long as vte (or oc) and only slightly longer than anterior ors; 4 ifr, none markedly enlarged, middle 2 pairs usually longer than posterior pair, foremost pair small, about ca. half the length of the previous pair; 1 microseta in front of anterior margin of frons, lateral to foremost ifr; 4–6 minute ads inside and below ors; g small, ca. as long as foremost peristomal setula and 1 or 2 short setae behind it; vi robust, ca. as long as vti; peristomal setulae (5–6) slightly longer than those in single postocular row; 3 postgenal setae, all relatively strong and curved. Frontal lunule of moderate length, well-developed, yellow and sparsely whitish microtomentose, slightly paler than anterior margin of frons. Face yellow, sparsely whitish microtomentose but facial cavities below antennae relatively shining; medial carina small, most distinct dorsally, below frontal lunule. Parafacialia darker than face, ochreous brown. Gena yellow, somewhat darkened only at vibrissal angle and very narrowly on ventral margin, all sparsely whitish microtomentose and rather dull. Postgena brown, sharply delimited from gena. Mouthparts ochreous to brownish including clypeus. Palpus yellowish, slender but distinctly clavate (Fig. 14), with ca. 5 dark setae (subapical longest) along ventral margin. Eye broadly suboval (9:8), of moderate size, with longest diameter ~ 6.0× as long as smallest genal height. Antenna brown (1^st^ flagellomere) to dark brown (scape and pedicel); 1^st^ flagellomere ca. as long as scape + pedicel, ellipsoid, with short greyish ciliation on apex (not longer than cilia on arista). Arista ~ 3.5× as long as antenna, shortly but densely ciliate.

***Thorax*** brown to pale brown (pleuron paler) and greyish brown microtomentose; mesonotum subshining, pleuron and scutellum more densely microtomentose and duller (Figs 12, 13). Mesonotum laterally (notopleural area) and posteriorly (in front of scutellum) paler, usually ochreous; scutellum also somewhat paler posteromedially. Thoracic pleuron with propleuron and sternopleuron largely pale brown to ochreous, other sclerites more or less ochreous margined. Scutellum large, transversely (8:5) rounded, trapezoidal, flat on disc. Thoracic chaetotaxy: mesonotal macrosetae relatively short and weak; 1 hu and 2 microsetae on humeral callus; 3 postsutural dc but the foremost very small (less than twice as long as dc microseta in front of it), the middle dc weak, ca. half the length of posterior, the latter long, ca. as long as scutellum; 6 rows of ac microsetae on suture; medial prescutellar ac pair distinctly prolonged, only slightly shorter than middle dc; 2 long sc, laterobasal ~ 1.3× as long as scutellum, apical (longest thoracic seta) ~ 1.4× as long as laterobasal; 2 stpl but anterior reduced to very small setula, sometimes indistinct.

**Figures 18–22.**
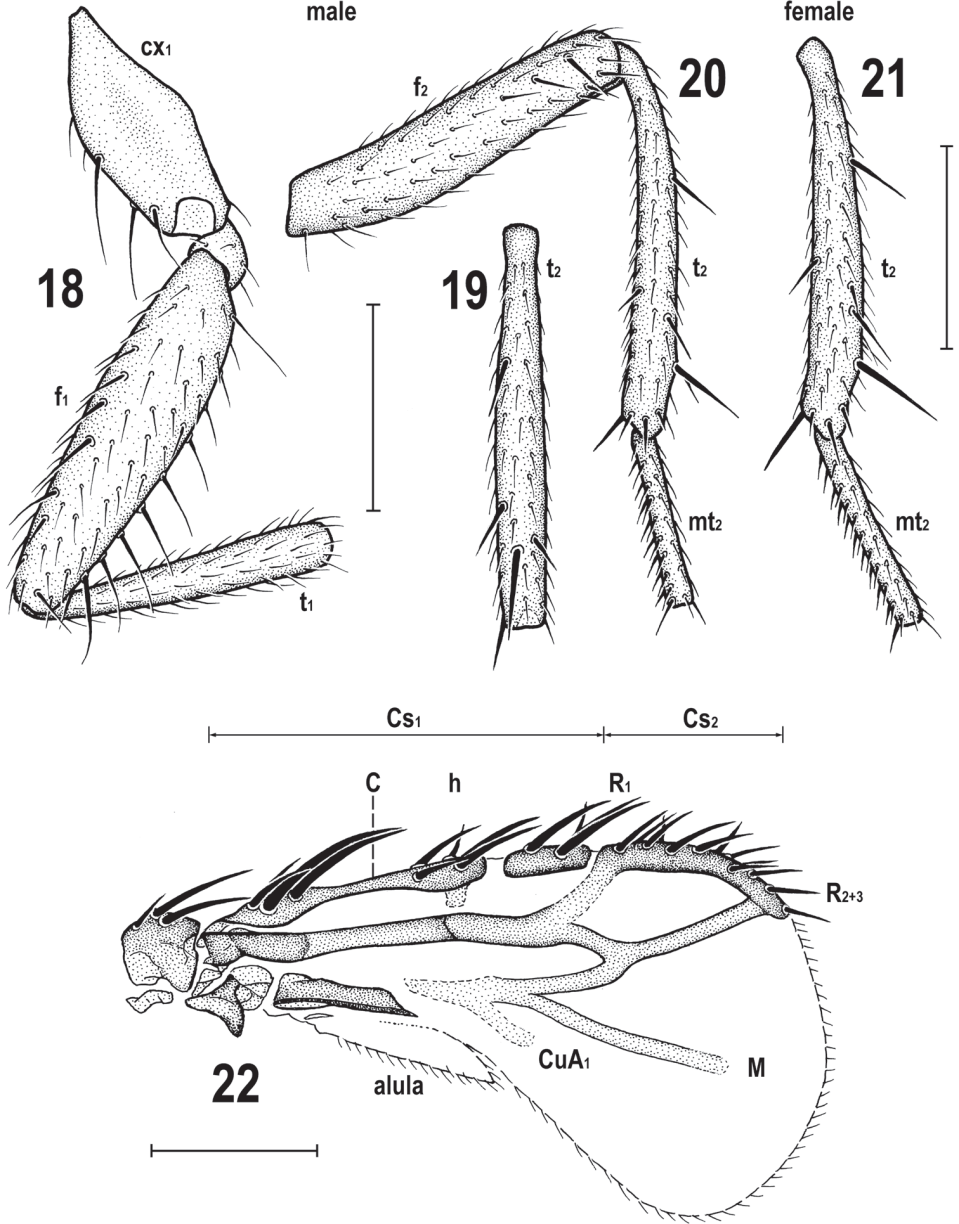
Pullimosina (Pullimosina) turfosa sp. nov. (paratypes) **18** male fore leg without tarsus, posteriorly **19** male mid tibia, dorsally **20** male mid femur, tibia and basitarsus, anteriorly **21** female mid tibia and basitarsus, anteriorly **22** male right wing, dorsally. Scale bars: 0.2 mm (**18–21**); 0.1 mm (**22**).

***Legs*** brown to pale brown, coxae, trochanters and knees ochreous to yellow; fore coxa and all trochanters lightest, dirty yellow. Chaetotaxy: f_1_ with a posterodorsal row of 6 or 7 shorter setae and a posteroventral row of 7 or 8 longer setae in addition to ventrobasal fine seta (Fig. 18). f_2_ ventrally uniformly setulose but with 3 anterodorsal setae in distal third, including longest subapical seta (Fig. 20). t_2_ (as in most European congeners) ventrally with 1 short and weak seta below middle (in distal two-fifths), 1 longer (but also relatively short) va seta and 1 small anteroapical seta (see Fig. 20); dorsally with only 4 setae, viz. 1 anterodorsal seta in proximal third, 1 anterodorsal seta in distal third, 1 long dorsal (most robust) seta in distal sixth and 1 small posterodorsal seta in distal fifth (Fig. 19). Hind leg, including f_3_, uniformly setulose. Ratio t_2_: mt_2_ = 2.17–2.30 (holotype 2.17).

***Wing*** (Figs 15, 22) strongly reduced, only ca. twice as long as scutellum, racket-shaped, with brownish membrane, most darkened around R_2+3_ and M; veins brown- to pale-pigmented. Distal radial and anal part of wing strongly reduced, thus R_4+5_ and A_1_ entirely absent. Basal part of C (= Cs_1_) well developed, including both breaks; distal part of C abbreviated (only Cs_2_ developed) so that C only slightly produced beyond apex of R_2+3_. Subcosta absent but presence of humeral (h) cross-vein indicated by darkened stump in front of humeral break (Fig. 22). Basal stem of radial veins robust but R_1_ short, pale pigmented and poorly visible (Fig. 15); R_2+3_ dark brown, very slightly to distinctly upcurved to C. M present, dark brown, forming anterior remnant of discal cell (Figs 15, 22); CuA_1_ strongly reduced, only indicated by a darkening near base of M. Anal lobe and hence also A_1_ absent; alula distinct but very narrow. Wing measurements: length 0.36–0.52 (holotype 0.52) mm, width 0.18–0.26 (holotype 0.26) mm, Cs_1_: Cs_2_ = 1.80–2.27 (holotype 1.80). Haltere present but strongly reduced (see Fig. 12), with knob entirely absent and stem shortened (length of haltere remnant 0.09–0.11 mm), dirty yellow.

**Figures 23–26.**
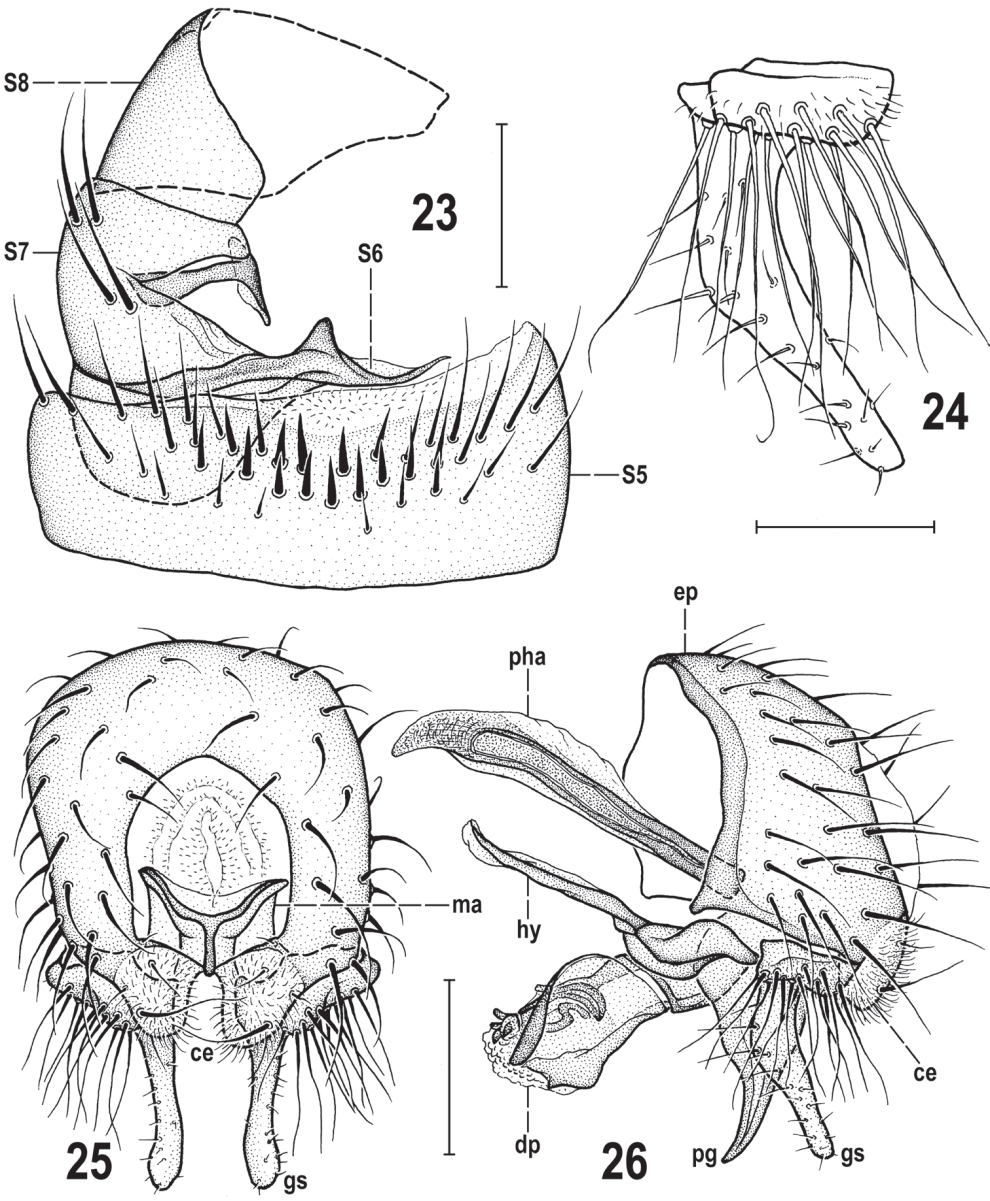
Pullimosina (Pullimosina) turfosa sp. nov. (male paratype) **23**S5 and postabdominal sclerites, ventrally **24** gonostylus, laterally **25** external genitalia, caudally **26** ditto, laterally. Scale bars: 0.1 mm (**23, 25, 26**); 0.05 mm (**24**). Abbreviations: ce – cercus, dp – distiphallus, ep – epandrium, gs – gonostylus, hy – hypandrium, ma – medandrium, pg – postgonite, pha – phallapodeme, S – sternum.

***Abdomen*** (Figs 12, 13) darker brown dorsally, paler (mainly anteriorly) brown ventrally. Preabdominal terga broad, transversely suboblong, and relatively shining because of sparse greyish brown microtomentum. T2–T5 sparsely but relatively long-setose, with longest setae in posterior corners and margins. T1+2 largest tergum, ~ 1.5× as long as T3, simply sclerotized (without medial depression) but original T1 pale brown to ochreous and distinctly delimited from original T2 (being dark brown) by a transverse wrinkle. T3‒T5 subequal in length but becoming slightly narrower posteriorly, T5 smallest. Preabdominal sterna: S1+2 small, reduced to pale and bare poorly delimited sclerite; S3 and S4 subequal in length, relatively large and broad (becoming wider posteriorly), brown and well-sclerotized; both S3 and S4 transversely trapezoidal, narrower anteriorly, but S3 distinctly smaller than S4, the latter smaller and narrower than S5. S3 and S4 with shorter and finer setae than adjacent terga. S5 (Fig. 23) darker brown than S3 or S4, more transverse, slightly asymmetrical (longer on left), with short posterior submembranous, unpigmented and finely haired margin and with a transverse group of robust setae, those in the middle particularly thickened, spine-like. Postabdominal sclerites S6+7 and S8 forming a relatively long complex synsclerite situated left ventrolaterally to dorsolaterally (Fig. 23). S6+7 strongly asymmetrical, with various projections and placed ventrolaterally to laterally; S8 less asymmetrical and situated more dorsally. Synsclerite S6+7 with original S6 attenuated right ventrally and bearing a distinctive subtriangular posteromedial (in medial axis of abdomen) process (Fig. 23), left ventrally dilated, without setae; original S7 ventrolaterally incised and with unusual slender T-shaped projection arising near this incision and directed right medially/internally (Fig. 23); left compact part of S7 with 2 pairs of relatively long and stout setae. S8 relatively simple, saddle-shaped, with only a few (3–5) shorter setae, mainly situated at posterior margin.

**Figures 27–30.**
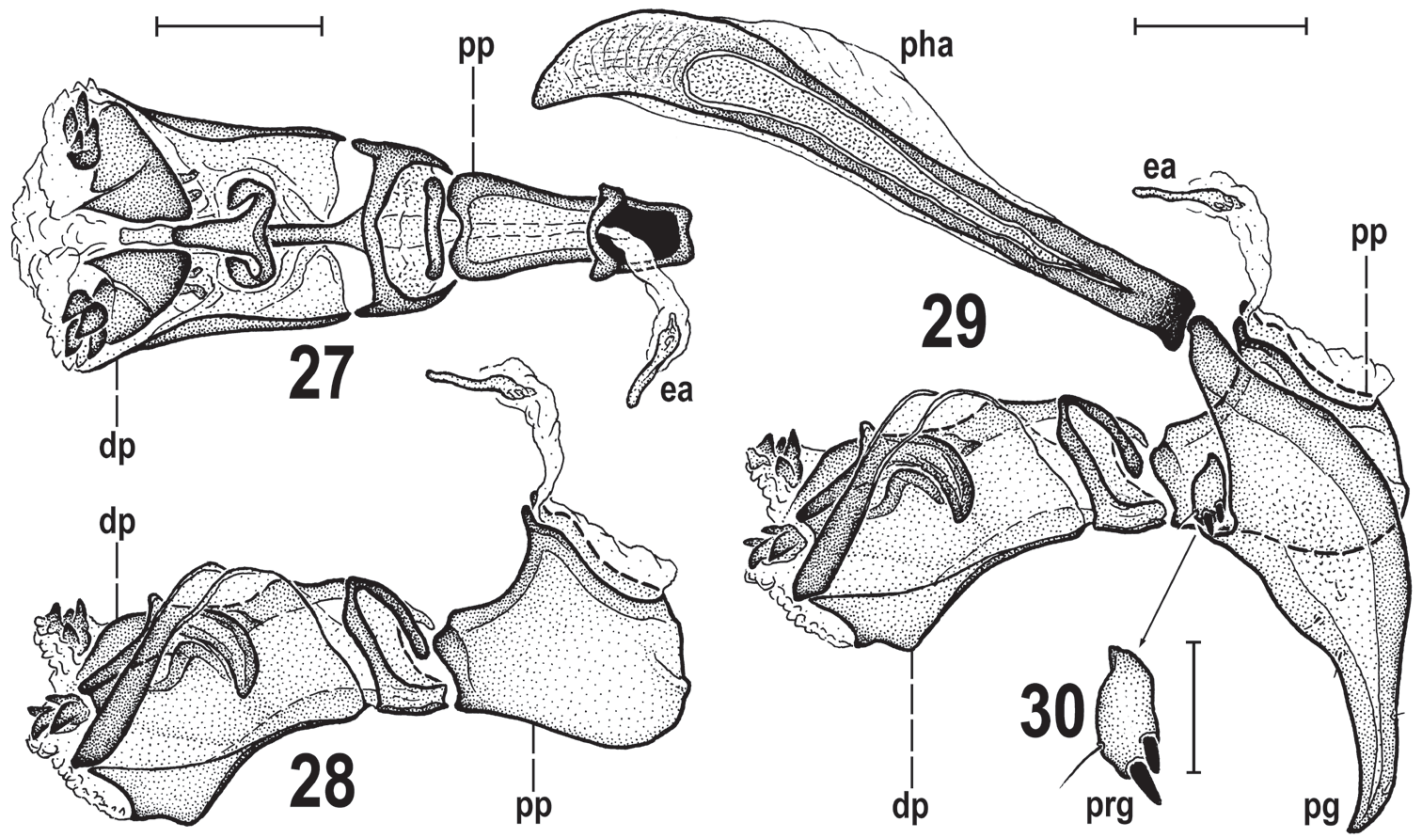
Pullimosina (Pullimosina) turfosa sp. nov. (male paratype) **27** aedeagus (phallus) dorsally **28** ditto, laterally **29** aedeagal complex, laterally **30** pregonite (enlarged), laterally. Scale bars 0.05 mm (27-29), 0.02 mm (30). Abbreviations: dp – distiphallus, ea – ejacapodeme, pg – postgonite, pha – phallapodeme, pp – phallophore, prg – pregonite.

***Genitalia***: Epandrium (Figs 25, 26) of medium length and width, very slightly asymmetrical in caudal view (Fig. 25), rather uniformly setose (longest setae postero­ventrally but sometimes also 1 dorsolateral seta enlarged). Anal fissure not large, roughly hexagonal, higher than wide (Fig. 25). Cerci short, fused with epandrium and medially forming subanal plate being ventromedially deeply narrowly incised (Fig. 25); each cercus with 1 longer and 2 or 3 short setae, micropubescent. Medandrium subquadrate in caudal view but its posterior part Y-shaped, hence ventrally narrowed (Fig. 25), posteromedially fused with cerci and posteroventrally movably connected with gonostyli. Hypandrium roughly Y-shaped in dorsal view, with simple anteromedial rod-like apodeme, relatively robust paired lateral sclerites, and more medially with small sclerites connecting hypandrium with postgonites via remnants of pregonites. Gonostylus (Figs 24–26) very distinctive, of unusual (in *Pullimosina*) shape: dorsally with small and low lateral part overgrown with a tuft of long sinuous setae and some micropubescence; anteroventrally (and more medially) protruding into a slender and long, slightly bent, apically blunt and shortly setulose projection. Aedeagal complex (Figs 27–30). Phallapodeme distinctly longer and more robust than hypandrial apodeme, with well-developed dorsal keel. Aedeagus composed of compact, laterally flattened phallophore (Figs 27, 28) and relatively short distiphallus. Distiphallus basally with slender arcuate sclerite bent on lateral sides (Figs 27, 28) and dilated ventrally; the latter dorsally connected with slender sclerite projecting anteriorly where bearing small wing-like processes and longer medial projection almost reaching apex of distiphallus (Fig. 27); distal part of distiphallus formed by large trough-like lateroventral sclerite and by a pair of apical dorsal sclerites, each of which having a group of 4 or 5 short dark spines attached laterally (Figs 27, 28). Postgonite (Fig. 29) relatively large (somewhat longer than distiphallus) but simple, wider proximally and gradually tapered distally, slightly bent and with acute apex, with only 2 or 3 microsetae anteriorly and posteriorly in distal half and fourth, respectively. Remnant of pregonite (Figs 29, 30) forming small but distinct and separate sclerite situated in anterodorsal emargination of postgonite, possessing distally 2 short blunt spines and 1 setula (see Fig. 30). Ejacapodeme reduced, represented by small and very slender, rod-like but proximally somewhat dilated, sclerite (Figs 27, 30).

**Figures 31–32.**
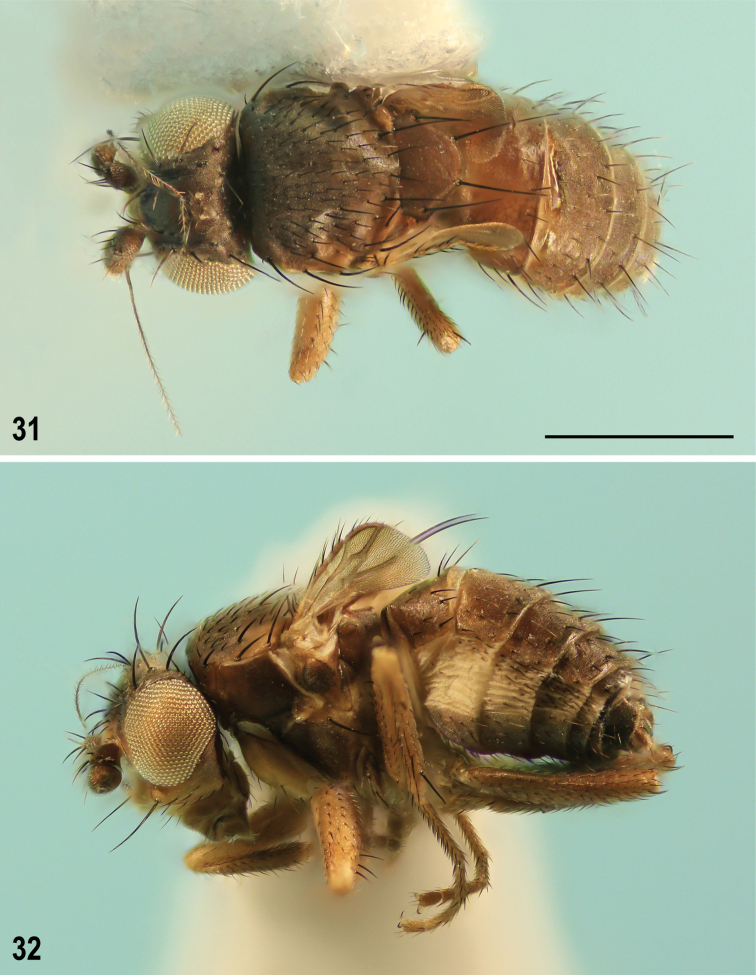
Pullimosina (Pullimosina) turfosa sp. nov. (female paratype) **31** whole body, dorsally **32** ditto, laterally. Scale bar: 0.5 mm.

**Female** (Figs 31, 32). Similar to male unless mentioned otherwise below. Total body length 1.27–1.67 mm. Foremost ifr more robust, often almost as long as other ifr setae. t_2_ with all macrosetae relatively longer, both ventrally (cf. ventroapical seta on Figs 20 and 21) and dorsally. mt_2_ relatively (compared to t_2_) longer (Fig. 21). Ratio t_2_: mt_2_ = 1.92–2.09. Wing (Fig. 17) slightly shorter on the average and often with more cut apex. Remnant of haltere also shorter, only 0.06–0.08 mm long. Wing measurements: length 0.36–0.43 mm, width 0.19–0.25 mm, Cs_1_: Cs_2_ = 2.14–3.00. Preabdominal terga somewhat shorter and more transverse (Fig. 32); T1+2 only slightly shorter than T3; T3–T5 becoming distinctly narrower posteriorly but similarly setose as in male. Preabdominal sterna S3–S5 sparsely and shortly setose, subequal in length and width. S5 unmodified, transversely suboblong, subequal to S4; preabdominal sterna S3–S5 brown, well sclerotized but paler than adjacent terga.

**Figures 33–39.**
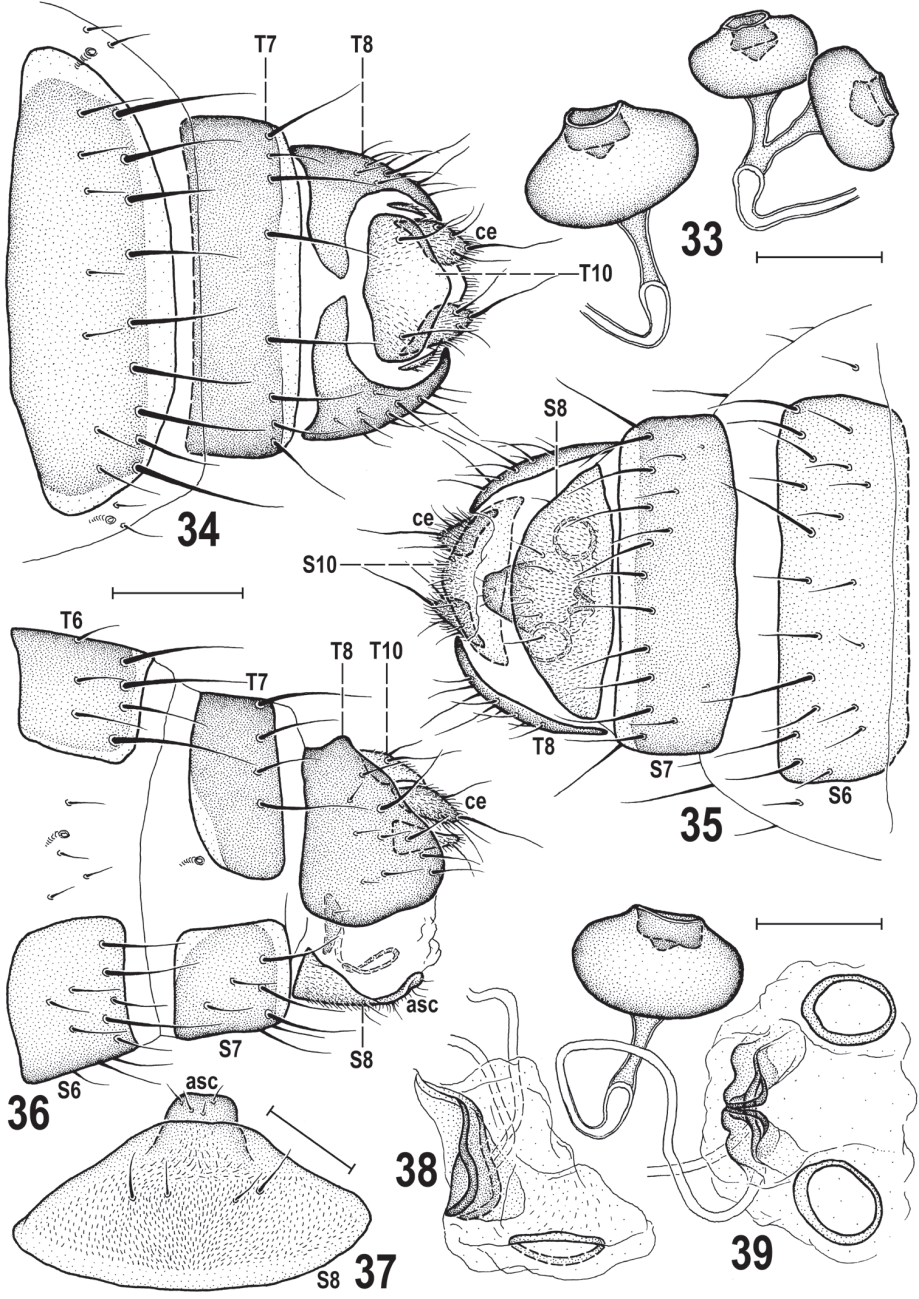
Pullimosina (Pullimosina) turfosa sp. nov. (female paratype) **33** spermathecae **34** postabdomen, dorsally **35** ditto, ventrally **36** ditto, laterally **37**S8 and additional sclerite, ventrally **38** spectacles-shaped sclerite, laterally **39** ditto and single spermatheca, ventrally. Scale bars: 0.1 mm (**34–36**); 0.05 mm (**33, 37–39**). Abbreviations: asc – additional sclerite, ce – cercus, S – sternum, T – tergum.

***Postabdomen*** (Figs 34–36) relatively short and broad, with sparsely setose sclerites, narrower than preabdomen at 5^th^ segment. T6 markedly narrower and only ca. half the length of T5, transverse, only slightly wider than S6, with both lateral and posterior margins pale and setose in posterior half (Fig. 34), setae at posterior margin long; T7 transversely suboblong, slightly shorter and seemingly narrower than T6 because bent farther onto lateral side (see Fig. 36), with pale posterior margin and 8 setae in single row of setae in front of it. T8 dorsomedially narrowly interrupted to form two lateral sclerites (Fig. 34), each dorsally shortened but ventrally expanded and longer than other postabdominal sclerites (Fig. 36) and bearing 1 long and a few short to small setae. T10 transversely pentagonal, distinctly wider than long, pale-pigmented, finely sparsely micropubescent and with a pair of relatively distant setae (see Fig. 34). S6 slightly narrower but distinctly (0.7× as long as) shorter than S5, and only slightly wider and more setulose than S7 (Fig. 35). S7 simple, transversely suboblong (as is S6), slightly wider than T6, with setae only at pale posterior margin. S8 (Figs 35, 37) transversely subellipsoid, much larger than S10 (in largest extension view, see Fig. 37), somewhat convex in the middle, posteriorly more rounded than anteriorly, with only 4 or 6 short setae centrally but with distinctive micropubescence. Additional sclerite unusual, situated behind and partly under S8 (its anterior part overlapped by S8, cf. Fig. 36, asc), narrowly trapezoidal but anteriorly membranous and hence its anterior margin undefined, largely bare, with only 4 setulae at posterior margin (Fig. 37). S10 slightly more than half length of S8, transversely pentagonal, pale pigmented, micropubescent and setulose only in posterior third, posteromedially with a pair of longer setae (Fig. 35). Spectacles-shaped sclerite (= sclerotization of female genital chamber) oriented rather vertically (Fig. 38, see in situ, Fig. 36), with rings of moderate size and its medial anterior sclerotization relatively complex (Fig. 39). Spermathecae 2+1 (Figs 33, 39), blackish brown; body of single spermatheca distinctly larger than those of paired ones; each spermatheca of relatively robust tyre-shaped form, most resembling those of *P.moesta* (Villeneuve, 1918), with plain surface, terminal invagination somewhat widened internally and terminal parts of ducts well-sclerotized, slightly conically dilated towards insertion and ca. as long as body of spermatheca. Cerci (Figs 34–36) short but not robust, tapered both towards base and terminal seta, micropubescent, each with 4 or 5 setae, apical one longest (slightly longer than cercus) and sinuate as also is the shorter dorsopreapical seta.

#### Remarks.

Despite a number of peculiarities in the male and female terminalia and unusual reduction of wing venation, *Pullimosinaturfosa* sp. nov. clearly is a representative of the subgenus Pullimosina s. str. (Roháček 1983; Marshall 1986). However, it proved not to be closely related to any other described European (or Palaearctic) species of this subgenus (cf. Papp 1973; Roháček 1983; Hayashi 2006; Su 2011; Su et al. 2013; Roháček 2019). Based on structures of its male and female terminalia it surely belongs to the *Pullimosinaantennata* group (as defined by Marshall 1986). Note: this group should be re-named to *P.moesta* group because *P.antennata* (Duda, 1918) is a junior synonym of *P.moesta* (Villeneuve, 1918), see Roháček (2001) and Roháček et al. (2001). *Pullimosinaturfosa* shares all synapomorphic characters defining this group (cf. Marshall 1986: fig. 100), viz. the densely and long setose gonostylus, the distiphallus with spinose or toothed distal sclerites and a well-developed additional sclerite between female S8 and S10, except for his character 11 (middle interfrontal setae cruciate). Surprisingly, *P.turfosa* appears to have the male terminalia most similar to those of the macropterous Nearctic species *P.vockerothi* Marshall, 1986. The shared characters include (1) male S6 with a ventromedial process (Fig. 23, cf. Marshall 1986: fig. 79), (2) the gonostylus with long and slender anteroventral projection (Fig. 24, cf. Marshall 1986: fig. 77), and (3) similar shape of postgonite (Fig. 30, cf. Marshall 1986: fig. 78). The former two features (1, 2) could be considered synapomorphic and demonstrating a closer relationship of these species. Additionally, the female T8 and the spectacles-shaped sclerite seem to be similarly formed in *P.vockerothi* and *P.turfosa* (Figs 34, 39, cf. Marshall 1986: figs 38, 40) but T8 in *P.vockerothi* has a small medial strip-like sclerite in addition to large lateral sclerites and the female S8 and additional (acs) sclerite are markedly different in the shape and chaetotaxy (Fig. 37, cf. Marshall 1986: fig. 39). There are also distinct differences in the armature of the male S5 (Fig. 23, cf. Marshall 1986: fig. 79), shape of the gonostylus (having basal part very small and anteroventral projection simple in *P.turfosa*: Fig. 24, cf. Marshall 1986: fig. 77) and detailed structure of the distiphallus (Fig. 28, cf. Marshall 1986: fig. 78).

*Pullimosinaturfosa* can be most easily recognized from all Holarctic *Pullimosina* species by its strongly abbreviated wings with very characteristic venation (Figs 15, 22). As for European species, the brachypterous form of the wing-polymorphic *P.meijerei* (Duda, 1918) externally most resembles this new species (cf. Roháček 2012: figs 3, 4) including coloration of the head but the wings of *P.meijerei* are less shortened, more elongate and with more complete venation (Roháček 2012: figs 24–27) not to mention very dissimilar structures of the male and female postabdomen (cf. Roháček 1985: figs 792–802).

The peculiar reduction of the wing and its veins in *P.turfosa* (Fig. 22) needs a special comment. It differs from all other cases of brachyptery known in West Palaearctic Sphaeroceridae (Roháček 2012) in having the distal part of wing strongly abbreviated while its basal part (up to subcostal break) is almost normal, R_4+5_ is completely absent (in this somewhat resembling the wing venation of *Aptilotusanapterus* (Papp & Roháček, 1981) from La Palma, Canary Is, which, however, has a small basal remnant of this vein retained) but simultaneously with M present. Thus, the reduction of veins in *P.turfosa* is somewhat intermediate between stages 5 and 6 as recognized by Roháček (2012: figs 39, 40).

#### Distribution.

The species is known only from its type locality in Russia, North Ossetia (Caucasus Mts).

#### Biology.

All specimens of the new species were collected on 17 and 18 August 2018 in a high-montane Chifandzar mire (Fig. 7), which is the highest (2289 m) and the largest (ca. 0.5 km^2^) of the mires under study. This mire is much more open and windier compared to the others.

All type specimens but one were collected from large *Sphagnum* hummocks (Fig. 8). This habitat is distinctive and represented only by nearly 15 hummocks all of which are located in the eastern part of the mire (Fig. 7: arrow). The hummocks are scattered over an area of ca. 100 × 50 m. Each hummock is 0.15–0.3 m high and 0.5–2 m wide. It consists mostly of loose thick cushion of *Sphagnum* (*S.teres* is predominant; *S.centrale* and *S.squarrosum* are common), with sparse shoots of *Carex* spp. and *Nardusstricta* and abundant remains of monocotyledons. The substrate of hummocks is dry to slightly wet, as distinct from moist or water-logged substrate on flat areas surrounding the hummocks and in other parts of the mire.

Most specimens were collected by means of sifting substrata of hummocks. Two females were collected by washing and subsequent flotation of substrate in NaCl solution: one specimen was sampled from the same habitat, and another one, from moist substrate beyond the hummocks, with predominating *Sphagnumsubsecundum* and *Carexrostrata*. Hence, most individuals of *P.turfosa* concentrate in hummocks but some flies may also occur at some distance from them. No specimens were collected in early summer (2–3 June), suggesting that the adults of *P.turfosa* appear later.

Due to exclusive association of *P.turfosa* with the sphagnetum habitat, particularly with hummocks, we consider it a tyrphobiont (= eucoenic to peat-bog habitat) sphagnicolous species. Interestingly, no specimens of *P.turfosa* were collected from similar substrata in other bogs using the same techniques (sifting and washing/flotation). It is possible that the new peculiar species is confined to high montane bogs or even endemic to Chifandzar, considering that the montane bogs of the North Caucasus are rare and isolated island ecosystems.

### ﻿Sphaeroceridae recorded from montane peat bogs in the North Caucasus

#### COPROMYZINAE (6 species)

*Borborillusuncinatus* (Duda, 1923)

*Borborillusvitripennis* (Meigen, 1830)

*Copromyzaequina* Fallén, 1820

*Lotophilaatra* (Meigen, 1830)

*Norrbomiacostalis* (Zetterstedt, 1847)

*Norrbomiasordida* (Zetterstedt, 1847)

#### SPHAEROCERINAE (1 species)

*Ischioleptanitida* (Duda, 1920)

#### LIMOSININAE (31 species)

*Chaetopodellascutellaris* (Haliday, 1836)

*Coproicaacutangula* (Zetterstedt, 1847)

*Coproicaferruginata* (Stenhammar, 1855)

*Coproicalugubris* (Haliday, 1836)

*Eulimosinaochripes* (Meigen, 1830)

*Gonioneuraspinipennis* (Haliday, 1836)

*Leptocerafontinalis* (Fallén, 1826)

*Leptoceranigra* Olivier, 1813

*Leptoceraoldenbergi* (Duda, 1918)

Minilimosina (Minilimosina) fungicola (Haliday, 1836)

Minilimosina (Minilimosina) gemella Roháček, 1983

Minilimosina (Minilimosina) sp.

Minilimosina (Svarciella) pujadei Carles-Tolrá, 2001

Minilimosina (Svarciella) vitripennis (Zetterstedt, 1847)

*Opacifronscoxata* (Stenhammar, 1855)

Opalimosina (Opalimosina) mirabilis (Collin, 1902)

Opalimosina (Pappiella) liliputana (Rondani, 1880)

Phthitia (Kimosina) longisetosa (Dahl, 1909)

Pseudocollinella (Spinotarsella) humida (Haliday, 1836)

Pullimosina (Pullimosina) heteroneura (Haliday, 1836)

Pullimosina (Pullimosina) turfosa sp. nov.

*Rachispodahostica* (Villeneuve, 1917)

*Rachispodalutosoidea* (Duda, 1938)

*Spelobiaclunipes* (Meigen, 1830)

*Spelobiaczizeki* (Duda, 1918)

*Spelobiaibrida* Roháček, 1983

*Spelobialuteilabris* (Rondani, 1880)

*Spelobiaparapusio* (Dahl, 1909)

*Spelobiarufilabris* (Stenhammar, 1855)

*Spelobiatalparum* (Richards, 1927)

*Terrilimosinaschmitzi* (Duda, 1918)

### ﻿Synopsis of species

#### COPROMYZINAE

***Borborillusuncinatus* (Duda, 1923) – TN**

**Material.** Kubus-larger, sweep-netting, 9.v.2016, 1♂.

**Comments.** A largely coprophagous species, widespread in temperate and northern belt of the Palaearctic Region. Its occurrence on a peat bog is surely occasional due to attraction to some mammal excrement. There is a single previous record from two bogs in Wales (Holmes et al. 1992)

***Borborillusvitripennis* (Meigen, 1830) – TN**

**Material.** Kubus-larger, sweep-netting, 9.v.2016, 1♂.

**Comments.** A coprophagous Palaearctic species, mainly associated with horse dung on pastures. There are only scarce records from peat bogs in England (Drake et al. 1989) and Estonia (Elberg 1971). It was captured together with the above species for the same reason.

***Copromyzaequina* Fallén, 1820 – TN**

**Material.** Kurnoyatsu-1, wet habitat (lake margin), sweep-netting, 6.vi.2018, 2♂.

**Comments.** A subcosmopolitan coprophagous species, occurring on (preferably horse) dung on pastures but also on manure. Also known to sometimes occur on peat bogs in Great Britain (Nelson 1971, 1981, 1982; Pitkin et al. 1985; Drake et al. 1989; Holmes et al. 1992) and Estonia (Elberg 1971), and was found on red-deer excrement on mires in the Czech Republic (Roháček 1984).

***Lotophilaatra* (Meigen, 1830) – TN**

**Material.** Kurnoyatsu-2, dry habitat (*Sphagnumfuscum*), sweep-netting, 7.vi.2018, 1♀; wet habitat (lake margin), 7.vi.2018, 1♀. Tarskoe, sweep-netting, 10.v.2016, 1♀; same but 1.vi.2018, 1♂; same but 11.ix.2018, 2♂.

**Comments.** A Holarctic, predominantly coprophagous, species, common on various animal dung. Although repeatedly recorded from several peat bogs in Europe (e.g., Peus 1928; Elberg 1969, 1971; Nelson 1971, 1981, 1982; Roháček 1984; Pitkin et al. 1985; Kuznetsova 1987; Drake et al. 1989; Holmes et al. 1992; Roháček and Barták 1999; Stuke and Roháček 2019) it has no closer affinity to peat-bog habitats because it is only attracted to various excrement, including droppings of voles (Roháček 1984).

***Norrbomiacostalis* (Zetterstedt, 1847) – TN**

**Material.** Chifandzar, sweep-netting 2 (daytime/sun), 18.vi.2018, 1 specimen (damaged, without abdomen); sweep-netting 1 (evening/rain), 17.ix.2018, 1♂ 1♀ (1♂ dry, genit. prep.).

**Comments.** A West Palaearctic coprophagous species, associated with (preferably horse) excrement on pastures. Formerly only recorded from a peat bog in Wales (Holmes et al. 1992).

***Norrbomiasordida* (Zetterstedt, 1847) – TN**

**Material.** Tarskoe, sweep-netting, 10.v.2016, 1♂ (dry, genit. prep.).

**Comments.** Originally a Holarctic (introduced to Neotropical, Oriental, and Oceanian Regions) coprophagous species, mainly occurring on dung of hoofed animals. There is no previous record from peat bogs.

#### SPHAEROCERINAE

***Ischioleptanitida* (Duda, 1920) – TPH**?

**Material.** Kurnoyatsu-2, wet habitat (lake margin), sweep-netting, 7.vi.2018, 1♂ (genit. prep.).

**Comments.** A Palaearctic saprophagous (mainly coprophagous) species preferentially occurring in humid places in open and woodland habitats at higher altitudes. It seems to have distinct affinity to bog habitats and was recorded from peat bogs in England (Nelson 1971), Central Europe (Pax 1937; Roháček 1984; Roháček and Barták 1999), and Latvia (Kuznetsova 1987). It was classified as a tyrphophilous species by Roháček (1984) and Roháček and Barták (1999).

#### LIMOSININAE

***Chaetopodellascutellaris* (Haliday, 1836) – TN**

**Material.** Tarskoe, sweep-netting, 11.ix.2018, 1♂.

**Comments.** A common, largely coprophagous species, widespread in the Palaearctic Region, most common on dung on pastures. Although it is known to occur on various peat bogs (Peus 1928; Rabeler 1931; Pitkin et al. 1985; Holmes et al. 1992; Roháček and Barták 1999), often on red-deer droppings (Roháček 1984) it was treated as a tyrphoneutral species both by Roháček (1984) and Roháček and Barták (1999).

***Coproicaacutangula* (Zetterstedt, 1847) – TN**

**Material.** Kurnoyatsu-1, dry habitat (*Sphagnummagellanicum*), sweep-netting, 6.vi.2018, 1♀; same but 22.ix.2018, 2♀.

**Comments.** A widespread (subcosmopolitan) coprophagous species associated with (mainly horse) dung on pastures. Records from bog habitats are very sporadic (Holmes et al. 1992; Stuke and Roháček 2019).

***Coproicaferruginata* (Stenhammar, 1855) – TN**

**Material.** Kurnoyatsu-2, wet habitat (lake margin), sweep-netting, 7.vi.2018, 1♂.

**Comments.** A cosmopolitan coprophagous species, very common on dung. Although repeatedly recorded from peat bogs and similar mire habitats in Europe (e.g., Nelson 1971; Doskočil 1973; Roháček 1984; Pitkin et al. 1985; Holmes et al. 1992; Roháček and Barták 1999; Stuke and Roháček 2019) it is classified as tyrphoneutral (Roháček 1984) because it is ubiquitous, occurring in any habitat with dung and other decaying matter.

***Coproicalugubris* (Haliday, 1836) – TN**

**Material.** Tarskoe, sweep-netting, 1.vi.2018, 1♀.

**Comments.** Another widespread (in the Palaearctic and Oriental Regions) coprophagous species, common on dung in pastures. It has been only occasionally recorded from peat bogs in Europe (Roháček 1984; Holmes et al. 1992; Roháček and Barták 1999; Stuke and Roháček 2019), mainly on excrement of ungulates.

***Eulimosinaochripes* (Meigen, 1830) – TN**

**Material.** Kurnoyatsu-2, wet habitat (lake margin), sweep-netting, 7.vi.2018, 1♀. Tarskoe, sweep-netting, 11.ix.2018, 1♀.

**Comments.** A Holarctic phytosaprophagous species, mainly living on meadows. There are records from lagg meadows of peat bogs in England (Nelson 1981), Wales (Holmes et al. 1992), Central Europe (Doskočil 1973; Roháček 1984), and Estonia (Elberg 1971), and, therefore, Roháček (1984) classified it as a tyrphoneutral species.

***Gonioneuraspinipennis* (Haliday, 1836) – TN**

**Material.** Kurnoyatsu-1, dry habitat (*Sphagnummagellanicum*), sweep-netting, 6.vi.2018, 1♀; same habitat, yellow pan traps, 6-8.vi.2018, 1♂; wet habitat (lake margin), sweep-netting, 6.vi.2018, 1♂; same but 7.vi.2018, 1♂. Kurnoyatsu-2, wet habitat (lake margin), sweep-netting, 7.vi.2018, 1♂. Chifandzar, wet habitat (*Sphagnumsubsecundum*), sample Ч 2, 3.vi.2018, 1♀. Tarskoe, sweep-netting, 10.v.2016, 1♂ 1♀.

**Comments.** A common Holarctic polysaprophagous species, mainly living on dung. In Central Europe it is relatively frequent on peat bogs (Doskočil 1973; Roháček 1984; Roháček and Barták 1999; Stuke and Roháček 2019) but was found on mires also in Britain (Nelson 1971; Pitkin et al. 1985; Holmes et al. 1992). This ubiquitous species is classified as tyrphoneutral (see also Roháček 1984).

***Leptocerafontinalis* (Fallén, 1826) – TN**

**Material.** Kurnoyatsu-1, dry habitat (*Sphagnummagellanicum*), sweep-netting, 6.vi.2018, 1♀; wet habitat (lake margin), sweep-netting, 6.vi.2018, 2♀. Kubus-smaller, sweep-netting (2), 14.ix.2018, 1♀.

**Comments.** A Holarctic saprophagous species, occurring in various humid habitat, mainly on decaying vegetation. Only Rabeler (1931); Nelson (1971); Pitkin et al. (1985); Drake et al. (1989), and Holmes et al. (1992) recorded it from peat bogs in Europe.

***Leptoceranigra* Olivier, 1813 – TN**

**Material.** Kurnoyatsu-2, wet habitat (lake margin), sweep-netting, 7.vi.2018, 1♂. Ushtulu, sweep-netting, 21.ix.2018, 1♀. Kubus-larger, sweep-netting, 4.vi.2018, 1♂; same but 14.ix.2018, 3♂ 2♀. Kubus-smaller, sweep-netting, 4.vi.2018, 1♂; same but 12.ix.2018, 2♂.

**Comments.** A very common species, widespread in the Old World but also introduced to Venezuela in the Neotropical Region (Buck and Marshall 2009). Polysapro­phagous, associated with open habitats. Roháček (1984) considered it tyrphoxenous but later on it has been recorded from several peat bogs in Central Europe (Roháček and Barták 1999; Stuke and Roháček 2019) and Great Britain (Holmes et al. 1992); therefore, it has been re-classified as tyrphoneutral (cf. Roháček and Barták 1999).

*Leptoceraoldenbergi (Duda, 1918) – TN

**Material.** Kurnoyatsu-1, dry habitat (*Sphagnummagellanicum*), sweep-netting, 6.vi.2018, 1♀ (genit. prep.). Kurnoyatsu-2, wet habitat (lake margin), sweep-netting, 7.vi.2018, 1♀.

**Comments.** A rare species known from temperate Europe (Belgium, Czech Republic, Denmark, Germany, Great Britain, Hungary, Ireland, Latvia, Netherlands, Slovakia, Sweden, Switzerland) but also recorded from Georgia (Roháček et al. 2001; Marshall et a. 2011). It is recorded for the first time from Russia. The species is usually found in undisturbed woodland habitats, often in runs of small mammals (Roháček 1982a) but hitherto unrecorded from peat bogs. Its surprising occurrence in Caucasian montane peat bogs could be due to presence of burrows of small mammals in bogs under study.

*Minilimosina (***Minilimosina***) ***fungicola* (Haliday, 1836) – T**N

**Material.** Kurnoyatsu-3, *Sphagnum*/sedge floating shores of lake, sweep-netting, 7.vi.2018, 2♀ (genit. prep.).

**Comments.** A Holarctic polysaprophagous species, habitat- and altitude-tolerant. It was recorded from mire habitats in England (Nelson 1971; Pitkin et al. 1985), Wales (Holmes et al. 1992), Central Europe (Roháček 1984; Roháček and Barták 1999; Stuke and Roháček 2019), and Latvia (Kuznetsova 1987). The above records seem to be the first from Russia (cf. Marshall et al. 2011).

*Minilimosina (Minilimosina) gemella Roháček, 1983 – TN?

**Material.** Kurnoyatsu-2, dry habitat (*Sphagnumfuscum*), sweep-netting, 7.vi.2018, 1♂ 1♀ (genit. prep.).

**Comments.** Also Holarctic, but essentially a Boreo-montane species, with a few records from Europe (cf. Marshall et al. 2011). First record from Russia. Although probably polysaprophagous as a larva, there is only a single previous record from peat bogs in Central Europe, viz. from the montane raised bog at Keprník-Vozka in the Hrubý Jeseník Mts, Czech Republic (Roháček 1984) but Pitkin et al. (1985) and Holmes et al. (1992) reported it from several bogs in England and Wales respectively.

***Minilimosina*** (***Minilimosina***) **sp.**

**Material.** Chifandzar, sweep-netting 2, 18.vi.2018, 1♀ (genit. prep.).

**Comments.** Based on structures of the female postabdomen, this specimen cannot be associated with any of *Minilimosina* (s. str.) species known from Europe. It could either be conspecific with some of the poorly characterized species described by Papp (1973, 1974) from Mongolia or belongs to an undescribed species. More material is necessary to solve the identity of this species.

*Minilimosina (***Svarciella***) ***pujadei* Carles-Tolrá, 2001 – TN**?

**Material.** Kubus-larger, sweep-netting, 9.v.2016, 1♂ (dry, genit. prep.).

**Comments.** Apart from the above new *Pullimosina* species, this species is the most surprising finding from the Caucasian peat bogs. It was described from a single male captured in a Malaise trap near a forest and a river at 1050 m in Andorra (Carles-Tolrá 2001) and subsequently recorded from the Podyjí National Park (Czech Republic: South Moravia), where another male was collected in pan traps installed in a forest-steppe habitat at only ca. 340 m (Roháček et al. 2005). The above finding (of a third known specimen) is the first from Russia and a new easternmost record of the species. Biology of *M.pujadei* remains unknown but considering the diversity of the recorded habitats (montane forest, forest-steppe, peat bog, Fig. 10) it seems to be an eurytopic, albeit extremely rare, species.

**Figures 40–43.**
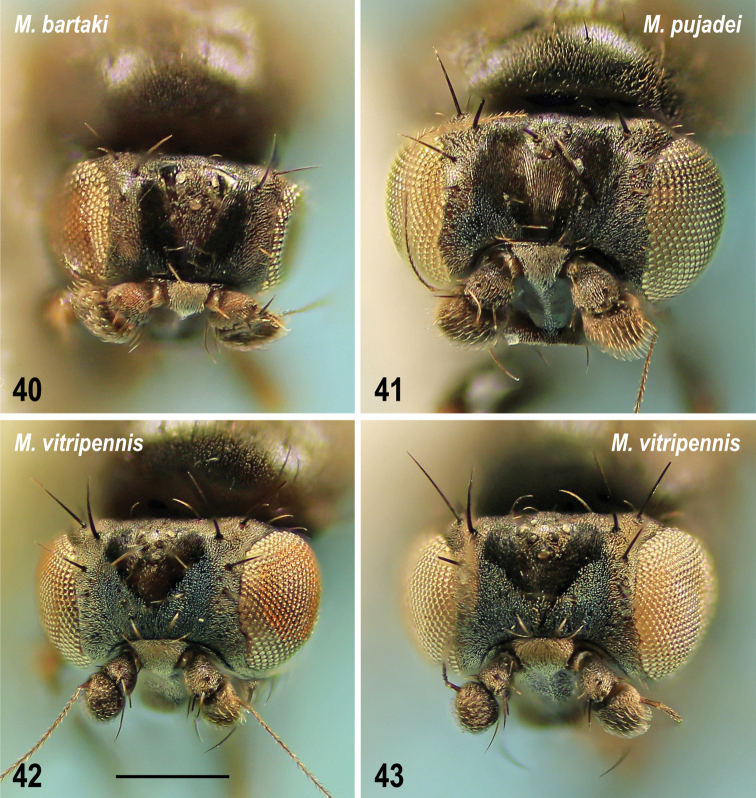
Heads of Minilimosina (Svarciella) species, frontally **40**M. (S.) bartaki Roháček, 2010, male paratype (Czech Republic) **41**M. (S.) pujadei Carles-Tolrá, 2001, male (Caucasus) **42**M. (S.) vitripennis (Zetterstedt, 1847), female (Czech Republic) **43** Ditto, female (Caucasus). Scale bar 0.2 mm.

**Taxonomic notes.** Owing to this surprising record, the Caucasian specimen has been compared to a male of *M.pujadei* from the Czech Republic (redescribed by Roháček 2010) including terminalia and pattern of frons. Conspecificity of both specimens has been confirmed. The heads of all three European species of the M. (S.) vitripennis group have been photographed to demonstrate distinct differences in their frontal microtomentose pattern. *Minilimosinapujadei* has the frons more similarly micro­tomentose to that of *M.bartaki* Roháček, 2010 while that of *M.vitripennis* differs from both these species in having frontal triangle smaller, forming a glabrous cordate spot surrounded by bluish silvery grey microtomentum (Figs 42, 43). *Minilimosinapujadei* (Fig. 41) can be distinguished from *M.bartaki* (cf. Fig. 40) by some bluish silvery grey microtomentum laterally at the anterior frontal margin and by fine longitudinal microsculpture medially in front of ocellar triangle (Fig. 41). Moreover, it also differs in having some microtomentum on the face (at least on medial carina and at ventral margin) while the face of *M.bartaki* is bare and shining.

***Minilimosina*** (***Svarciella***) ***vitripennis* (Zetterstedt, 1847) – TN**

**Material.** Kurnoyatsu-1, wet habitat (lake margin), sweep-netting, 6.vi.2018, 1♀ (dry, genit. prep.). Kurnoyatsu-2, dry habitat (*Sphagnumfuscum*), sweep-netting, 7.vi.2018, 1♂ (genit. prep.); same habitat, yellow pan traps, 7-8.vi.2018, 1♂ (genit. prep.). Kurnoyatsu-3, *Sphagnum*/sedge floating shores of lake, sweep-netting, 7.vi.2018, 1♂. Kubus-larger, sweep-netting, 4.vi.2018, 1♂ 2♀.

**Comments.** A Holarctic polysaprophagous species associated with various, mainly open, habitats (meadows, pastures etc.). There are also a few records from mire habitats in England (Nelson 1971, as *Leptocerafungicola*, Pitkin et al. 1985), Wales (Holmes et al. 1992), and Central Europe (Roháček 1984; Roháček and Barták 1999; Stuke and Roháček 2019).

**Figures 44–45.**
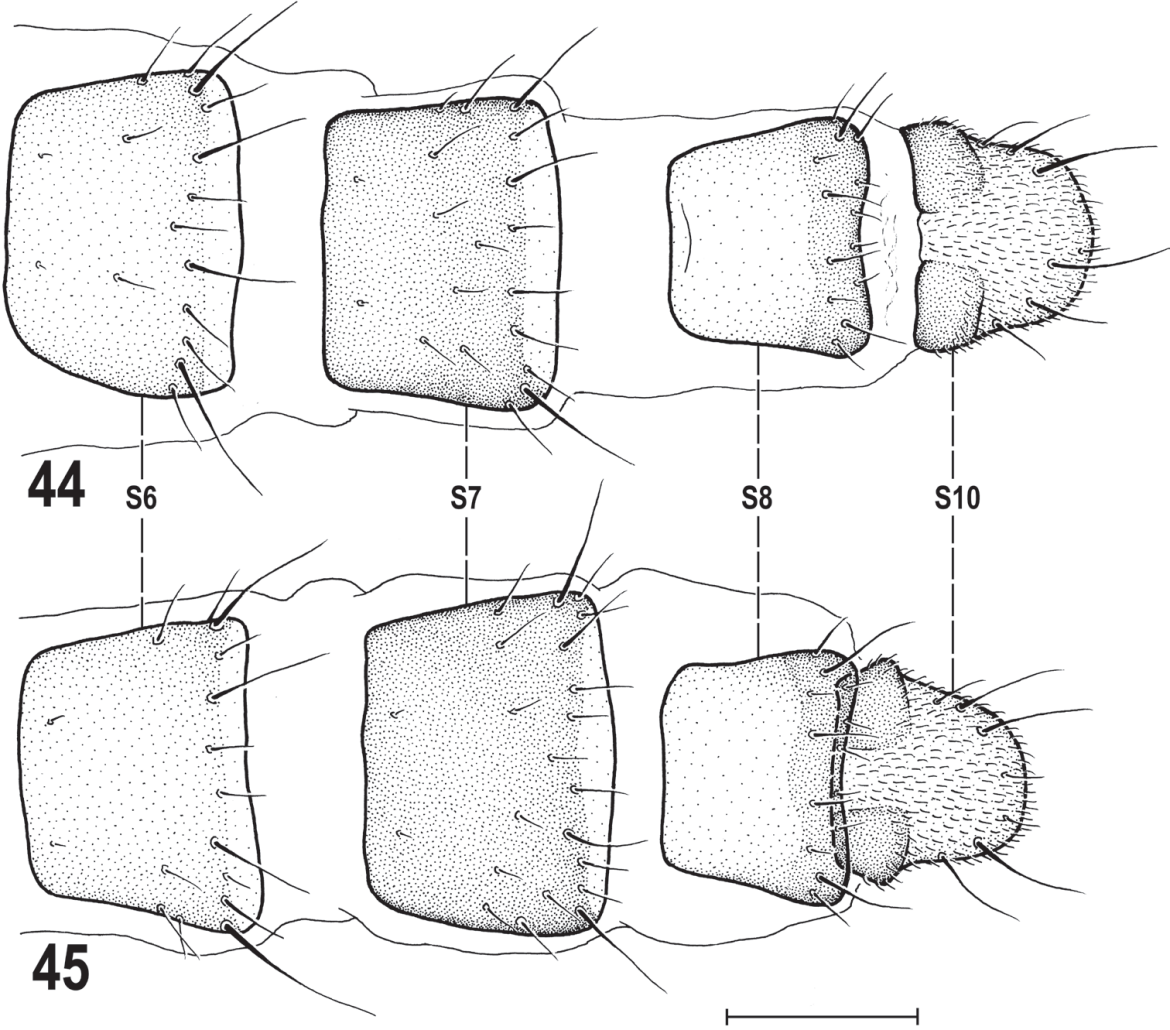
Minilimosina (Svarciella) vitripennis (Zetterstedt, 1847), female postabdominal sterna, ventrally **44** female from the Czech Republic **45** female from the Caucasus. Scale bar 0.1 mm. Abbreviations: S – sternum.

**Taxonomic notes.** A female with enlarged glabrous frontal triangle (Fig. 43) similar to that described for the holotype of *Limosinaparavitripennis* Papp, 1973 (Mongolia), has been also studied for postabdominal structures and compared to those of typical specimens of M. (S.) vitripennis from other parts of Europe. This examination resulted in finding that although this female from the Caucasus surely is conspecific (see below) with other European specimens, the ventral postabdominal sclerites (S8 and S10 in particular) seem to differ from those figured for this species in the revision by Roháček (1982b: fig. 190). The latter illustration proved to be somewhat simplified and, therefore, the postabdominal sterna of *M.vitripennis* are here newly illustrated and described for both Caucasian (Fig. 45) and the Czech specimens (Fig. 44): S8 is distinctly transversely darkened at posterior margin (Figs 44, 45) while its large anterior part is pale-pigmented; S10 (subanal plate) (Figs 44, 45) is distinguished by angularly separated and darkened anterolateral areas, while its main (posterior) part is posteriorly rounded and densely micropubescent; this micropubescence is also expanded anteromedially, between darkened anterolateral areas; S10 is otherwise with several marginal setae, 2 pairs of them are long, often longer than the longest setae on S7 and S8. True, the female from the Caucasus has S10 more angular anterolaterally (Fig. 45) but this aberration is considered to fall within the variability of *M.vitripennis* as is its enlarged frontal triangle. Roháček and Marshall (1988) reached the same conclusion when they synonymized *Limosinaparavitripennis* Papp, 1973 with M. (S.) vitripennis.

***Opacifronscoxata* (Stenhammar, 1855) – TN**

**Material.** Kurnoyatsu-1, wet habitat (lake margin), sweep-netting, 6.vi.2018, 1♀; same habitat, yellow pan traps, 6-8.vi.2018, 1♂. Kurnoyatsu-2, wet habitat (lake margin), sweep-netting, 7.vi.2018, 1♀. Ushtulu, sweep-netting, 21.ix.2018, 1♂.

**Comments.** A common Palaearctic species associated with mud on shores of water bodies and in marshland habitats. It is eurytopic and, therefore, able to live on peat mud in mires (see Rabeler 1931; Doskočil 1973; Roháček 1984; Pitkin et al. 1985; Kuznetsova 1987; Drake et al. 1989; Holmes et al. 1992; Roháček and Barták 1999; Stuke and Roháček 2019). Due to its occurrence in various muddy habitats it is considered a tyrphoneutral species.

***Opalimosina*** (***Opalimosina***) ***mirabilis* (Collin, 1902) – TN**

**Material.** Kurnoyatsu-2, wet habitat (lake margin), sweep-netting, 7.vi.2018, 1♀.

**Comments.** A subcosmopolitan ubiquitous, predominantly coprophagous species. It is extremely habitat-tolerant and, therefore, can also occur on various excrement on peat bogs; see e.g., Nelson (1971); Doskočil (1973); Roháček (1984); Holmes et al. (1992); Roháček and Barták (1999), and Stuke and Roháček (2019).

***Opalimosina (Pappiella) liliputana* (Rondani, 1880) – TN**

**Material.** Kurnoyatsu-1, wet habitat (lake margin), sweep-netting, 6.vi.2018, 1♂ 1♀ (genit. prep.). Kurnoyatsu-2, dry habitat (*Sphagnumfuscum*), sweep-netting, 7.vi.2018, 1♂.

**Comments.** A Holarctic and widely habitat-tolerant species developing in various decaying substrates. In Europe, it was sporadically recorded also from mire habitats, viz. by Nelson (1971); Roháček (1984); Pitkin et al. (1985); Holmes et al. (1992), and Roháček and Barták (1999).

***Phthitia (Kimosina) longisetosa* (Dahl, 1909) – TPH**?

**Material.** Chifandzar, dry habitat (*Sphagnumteres* hummocks), sifting, 18.ix.2018, 1♀ (genit. prep.).

**Comments.** A rather rare West Palaearctic species with poorly known biology, usually occurring in damp meadows, fens and humid forests, sometimes in burrows of small mammals (cf. Roháček 1983), in Russia only known from an old record from West Siberia (Duda 1938). Interestingly, the species was described from a single female captured in “Torfmoos” (= peat moss) in a raised bog in Germany (Dahl 1909). Peus (1928, 1932) and Stuke and Roháček (2019) also recorded it from raised peat bogs in Germany, Pitkin et al. (1985) from peatlands in England and Holmes et al. (1992) found it fairly frequent in Welsh peatlands. Consequently, although hitherto unknown from peat bogs in the Czech Republic (Roháček 1984; Roháček and Barták 1999), this species may have a distinct affinity to bog habitats (as suggested by Peus 1932). The above specimen (peculiar for its unusually small size: body length only 1.5 mm) has been sifted from *Sphagnumteres* hummocks together with the type series of *Pullimosinaturfosa* sp. nov.

***Pseudocollinella (Spinotarsella) humida* (Haliday, 1836) – TN**

**Material.** Kurnoyatsu-2, wet habitat (lake margin), sweep-netting, 24.ix.2018, 1♀. Kubus-smaller, sweep-netting, 4.vi.2018, 1♀.

**Comments.** A common paludicolous species, widespread in the Palaearctic Region. It is associated with muddy habitats and is also known to live on peat bogs in Europe (Elberg 1971; Nelson 1982; Nelson and Theaker 1982; Roháček 1984; Drake et al. 1989; Holmes et al. 1992; Roháček and Barták 1999; Stuke and Roháček 2019) although peat mud surely is not its preferred habitat.

***Pullimosina*** (***Pullimosina***) ***heteroneura* (Haliday, 1836) – TN**

**Material.** Kurnoyatsu-1, wet habitat (lake margin), sweep-netting, 6.vi.2018, 1♂. Tarskoe, 10.v.2016, sweep-netting, 1♀.

**Comments.** A cosmopolitan, eurytopic polysaprophagous species, common on various rotting substrates but records (of only single specimens) from peat bogs are scarce (Roháček 1984; Pitkin et al. 1985; Holmes et al. 1992; Roháček and Barták 1999; Stuke and Roháček 2019).

*Pullimosina (***Pullimosina***) ***turfosa* sp. nov. – TB**

**Material.** Chifandzar, dry habitat (*Sphagnumteres* hummocks), sample Ч 14, 17.ix.2018, 1♀; same habitat, sifting, 18.ix.2018, 6♂ 5♀; wet habitat (*Sphagnumsubsecundum*), sample Ч 9, 17.ix.2018, 1♀ (see also type material above; some with genit. prep.)

**Comments.** This new terricolous and strongly brachypterous species (described above) is surely tyrphobiont, i.e., exclusively associated with peat-bog habitat, because living in *Sphagnum* hummocks (Fig. 8). Its habitat is thus very similar to that of the circumboreal Boreo-montane tyrphobiont species Pullimosina (Dahlimosina) dahli (Duda, 1918) (cf. Roháček 1984, Roháček and Barták 1999). Because the latter species nor any other tyrphobiont species of Sphaeroceridae known from Europe (see Roháček and Barták 1999) have been found in the peat bogs under study, *P.turfosa* sp. nov. is currently the only true tyrphobiont sphaerocerid recorded from the Caucasus.

***Rachispodahostica* (Villeneuve, 1917) – TX**?

**Material.** Kurnoyatsu-2, wet habitat (lake margin), sweep-netting, 7.vi.2018, 1♀ (genit. prep.).

**Comments.** An uncommon Palaearctic species (but unrecorded from most of Western Europe and North Africa) with easternmost records from Mongolia (Roháček 1991) and known from the Caucasus (Papp 1979). Adults occur on mud on shores or in marshy habitats (also in mountains) but there is no previous record from peat bogs. Consequently, we consider the above record rather exceptional.

***Rachispodalutosoidea* (Duda, 1938) – TN**

**Material.** Tarskoe, sweep-netting, 30.v.2018, 1♂.

**Comments.** A common West Palaearctic species (also recorded from the Caucasus, cf. Roháček 1991) living on mud in various open and woodland habitats. In contrast to related and more eurytopic *R.lutosa* (Stenhammar, 1855) it seems to occur on peat mud rarely: there is only one previous record from a montane peat bog in the Czech Republic (Roháček 1984, 1991).

***Spelobiaclunipes* (Meigen, 1830) – TN**

**Material.** Kurnoyatsu-1, wet habitat (lake margin), sweep-netting, 6.vi.2018, 2♀. Kurnoyatsu-2, dry habitat (*Sphagnumfuscum*), sweep-netting, 7.vi.2018, 3♀; wet habitat (lake margin), sweep-netting, 7.vi.2018, 2♀; wet *Sphagnum* habitat (lake margin), yellow pan traps, 7-8.vi.2018, 2♂. Kurnoyatsu-3, *Sphagnum*/sedge floating shores of lake, sweep-netting, 7.vi.2018, 1♂ 2♀ (genit. prep.). Kubus-larger, sweep-netting, 4.vi.2018, 2♂; sweep-netting (2), 14.ix.2018, 1♀. Tarskoe, sweep-netting, 10.v.2016, 6♂ 5♀ (genit. prep.).

**Comments.** A very common, eurytopic and polysaprophagous Holarctic species. Because of its wide habitat and trophic tolerance, it was often recorded from peat bogs in Europe (e.g., Krogerus 1960; Nelson 1971; Doskočil 1973; Roháček 1984; Pitkin et al. 1985; Drake et al. 1989; Holmes et al. 1992; Roháček and Barták 1999; Stuke and Roháček 2019). It has also been frequently encountered in peat bogs in the Caucasus.

***Spelobiaczizeki* (Duda, 1918) – TN**

**Material.** Kurnoyatsu-1, dry habitat (*Sphagnummagellanicum*), sweep-netting, 6.vi.2018, 1♂ (genit. prep.). Kurnoyatsu-3, *Sphagnum*/sedge floating shores of lake, sweep-netting, 7.vi.2018, 1♀.

**Comments.** A rather uncommon polysaprophagous Palaearctic species associated with various subterranean habitats (Roháček 1983). It has not been previously recorded from peat bogs. Its finding in the Caucasian peat bogs is surely due to the presence of burrows of small mammals in the localities listed above.

*Spelobiaibrida Roháček, 1983 – TPH

**Material.** Kubus-larger, sweep-netting, 14.ix.2018, 1♀. Kubus-smaller, sweep-netting, 14.ix.2018, 1♂ (genit. prep.).

**Comments.** An uncommon phytosaprophagous species known from montane regions of Central and South Europe (Roháček et al. 2001) but also reported from peatlands in Canada (Marshall 1994). First records from Russia. The species was recorded from wet montane forests (Roháček 1983) and from a few peat bogs (Roháček 1983, 1984; Roháček and Barták 1999) in the Czech Republic and is, therefore, treated as tyrphophilous by the latter authors.

***Spelobialuteilabris* (Rondani, 1880) – TN**

**Material.** Kurnoyatsu-2, dry habitat (*Sphagnumfuscum*), sweep-netting, 7.vi.2018, 1♂ (genit. prep.).

**Comments.** A polysaprophagous, habitat- and altitude-tolerant, originally Holarctic species. It is also known from some European peat bogs (Nelson 1971; Doskočil 1973; Roháček 1984; Pitkin et al. 1985; Holmes et al. 1992; Roháček and Barták 1999).

***Spelobiaparapusio* (Dahl, 1909) – TN**

**Material.** Kurnoyatsu-2, dry habitat (*Sphagnumfuscum*), yellow pan traps, 22–24.ix.2018, 1♂ (genit. prep.). Kubus-smaller, yellow pan traps, 12-14.ix.2018, 1♂. Tarskoe, sweep-netting, 11.ix.2018, 1♂.

**Comments.** An originally Palaearctic mycophagous species developing in sporocarps of various terrestrial macrofungi, mainly in woodland. It is parthenogenetic in Central and North Europe but bisexual in southern parts of the Palaearctic Region. *Spelobiaparapusio* was described from 1♀ collected in *Sphagnum* on a raised bog in Germany (Dahl 1909) and subsequently recorded from several peat bogs (mostly on fungi) in the Czech Republic (Roháček 1984; Roháček and Barták 1999) and Latvia (Kuznetsova 1987). It is peculiar that in the Caucasian peat bogs under study only males have been captured.

***Spelobiarufilabris* (Stenhammar, 1855) – TN**

**Material.** Kubus-smaller, sweep-netting (1), 12.ix.2018, 1♀; same bog, yellow pan traps, 12-14.ix.2018, 1♂ (genit. prep.).

**Comments.** A phytosaprophagous Palaearctic species living mainly in colder and humid montane forests but (less frequently) also occurring on peat bogs (Elberg 1969, 1971; Nelson 1971; Roháček 1984; Pitkin et al. 1985; Holmes et al. 1992; Roháček and Barták 1999; Stuke and Roháček 2019). First record from southern European Russia (cf. Marshall et al. 2011).

***Spelobiatalparum* (Richards, 1927) – TN**

**Material.** Kubus-smaller, sweep-netting (1), 12.ix.2018, 1♀ (genit. prep.).

**Comments.** A common microcavernicolous species, widespread in the Palaearctic Region. It is polysaprophagous and inhabits subterraneous nests and runs of mammals. There are several records from peat bogs in the Czech Republic (Roháček 1984; Roháček and Barták 1999), in England (Pitkin et al. 1985), and in Wales (Holmes et al. 1992). Roháček (1984) mentioned regular occurrence of this species in runs of *Microtusagrestis* in this habitat. The above record from a montane bog in the Caucasus indicates the presence of voles in this locality.

***Terrilimosinaschmitzi* (Duda, 1918) – TN**

**Material.** Kubus-smaller, sweep-netting, 4.vi.2018, 1♂ (genit. prep.).

**Comments.** A widespread Holarctic species associated with forest litter in humid montane woodland. It was also ascertained in peat bogs in Wales (Holmes et al. 1992), the Czech Republic (Roháček 1984; Roháček and Barták 1999), and Latvia (Kuznetsova 1987), usually in marginal parts of bogs.

## ﻿Discussion and conclusions

A total of 38 species of Sphaeroceridae has been found in eight montane and submontane mires of the North Caucasus. Sphaeroceridae appeared to be rather diverse but not abundant in the insect fauna of these mires, as compared to members of many other dipteran families, which were much more common. Extensive sampling with seven main techniques provided only 119 specimens of Sphaeroceridae. Most Sphaero­ceridae species (31) were represented only by 1–3 specimens; most species (32) were found only in one or two mires. Only one species, *Spelobiaclunipes*, was relatively common and, at the same time, it was recorded from the highest number of mires (5 of 8). The highest species number (17) was recorded in the habitat-diverse Kurnoyatsu-2, the lowest (2–5), in the most uniform and largest fens of Ushtulu and Chifandzar and in a relatively uniform small mire Kurnoyatsu-3.

Because Sphaeroceridae are usually partly or wholly neglected in studies on insect communities of peat bogs (see Introduction), the above results obtained on the Caucasian mires can only be compared with those from more detailed investigations of this family in the Czech Republic (Roháček 1984; Roháček and Barták 1999) and Wales (Holmes et al. 1992), and (partly, because of limited data) from England (Nelson 1971; Pitkin et al. 1985) and Latvia (Kuznetsova 1987). The fauna of Sphaeroceridae in Central European peat bogs seems to be much more diverse than the Caucasian ones. Roháček (1984) recorded as many as 80 species from nine peat bogs in the Hrubý Jeseník and Králický Sněžník Mts in northern Moravia and Roháček and Barták (1999) recorded 66 species from 14 peat bogs in the Šumava Mts in southern Bohemia (both in the Czech Republic). The peatland survey of Sphaeroceridae in Wales (Holmes et al. 1992) recorded as many as 78 species but this research also included poor and rich fens, not only raised bogs. Thus, the number of species in the Caucasian mires resembles those recorded from mire habitats in England (Nelson 1971: 31 species; Pitkin et al.1985: 26 species) but is distinctly higher than in two raised bogs in Latvia (Kuznetsova 1987: 17 species). However, it should be stressed that the research of Diptera on peat bogs in the Czech Republic was much more intensive (performed during several years from May/June to September/October by two-weekly to monthly sampling) and the material collected was more numerous. Therefore, the mere comparison of the number of species recorded in the above studies is somewhat misleading and the actual species richness of sphaerocerid flies in bogs under study would be much larger if they were sampled for Diptera during the entire warm season and repeatedly for more years. The qualitative comparison of taxa with distinct affinities to bog habitats is a more important signal to evaluate similarity / dissimilarity of local faunas of Sphaeroceridae in peat bogs.

### ﻿Tyrphobiont species

In the Caucasian mires under study only one (and new) tyrphobiont species was found, Pullimosina (P.) turfosa sp. nov. In Europe, three other species have been formerly placed in this category: two circumpolar (hence Holarctic) Boreo-montane species of Limosininae, Pullimosina (Dahlimosina) dahli (Duda, 1918) and *Spelobiapappi* Roháček, 1983, and one species of Copromyzinae hitherto only known from raised bogs in the Czech Republic and Austria, *Crumomyiatyrphophila* Roháček, 1999 (see Roháček and Barták 1999). Considering the presence of tyrphobiont species of Sphaeroceridae, the Caucasian mires seems to be dissimilar to those in Central and North Europe which share at least *P.dahli* and *S.pappi*.

### ﻿Tyrphophilous species

The following ten species have been classified as tyrphophilous by Roháček (1984) and Roháček and Barták (1999) based on studies of peat-bog fauna in the Czech Republic: *Copromyzaneglecta* (Malloch, 1913) and *C.stercoraria* (Meigen, 1830) (Copromyzinae), *Ischioleptanitida* (Duda, 1920) (Sphaerocerinae), *Minilimosinaguestphalica* (Duda, 1918) (= *M.v-atrum* auctt.), Phthitia (Collimosina) spinosa (Collin, 1930), *Pteremisfenestralis* (Fallén, 1820), Pullimosina (Pullimosina) pullula (Zetterstedt, 1847), *Spelobiabelanica* Roháček, 1983, *S.ibrida* Roháček, 1983, and *S.nana* (Rondani, 1880). Of these only two, viz. *I.nitida* and *S.ibrida*, have been recorded from mires in the North Caucasus but it is suggested above that a third species, Phthitia (Kimosina) longisetosa (Dahl, 1909), is also to be included in the tyrphophilous category, despite being hitherto unrecorded from peat bogs in the Czech Republic. However, there are several other tyrphophilous candidates in Sphaeroceridae (Limosininae), particularly *Spelobiabispina* Marshall, 1985, a circumpolar Boreal species known from peatlands in Canada (Marshall 1994) and from Sweden (Florén 1989) and the Baikal area in eastern Siberia (Roháček et al. 2001). *Pseudocollinellaabhorrens* (Roháček, 1990) could be a similar case, occurring in the Canadian tundra and some more southern peatlands in Canada and USA (Marshall 1994) and in Sweden (Roháček 1990). Minilimosina (Minilimosina) tenera Roháček, 1983, a species described from a single male from the peat bog Skřítek in northern Moravia (Czech Republic) and subsequently recorded from an upland mire in Wales (Holmes et al. 1992), a wet coniferous forest in Sweden (Florén 1989), and near a subarctic forest spring in Finland (Haarto and Kahanpää 2013), could also belong to this category. Thus, up to 14 species can be classified as tyrphophilous in European peat bogs but only three of them have been recorded from montane and submontane mires in the North Caucasus.

### ﻿Tyrphoneutral species

The vast majority (34 species) of Sphaeroceridae recorded from Caucasian mires belong to this category which is in agreement with the situation in Central and North European bogs. This group is mainly composed of common eurytopic coprophagous or saprophagous species, which are able to survive (and even prosper) in peat bogs if there is enough food supply for their larvae (animal excrement and carrion and decaying vegetation). Five of the eight mires under study can provide favorable conditions to many coprophagous species of Sphaeroceridae as their environs and essentially the bogs are used for grazing horses or cattle (see “Localities under study” for further details). *Borborillus*, *Copromyza*, *Lotophila*, *Norrbomia*, *Chaetopodella*, *Coproica*, *Gonioneura*, and *Opalimosina* species are typical examples of coprophages while *Eulimosina*, *Leptocera*, *Minilimosina*, *Pullimosina*, and most *Spelobia* and *Terrilimosina* species are phytosaprophagous or polysaprophagous. The herein recorded paludicolous *Opacifrons*, *Pseudocollinella*, and *Rachispoda* species are also eurytopic and can develop in the acidic mud in these mires, perhaps with the exception of *Rachispodahostica* (Villeneuve, 1917) being obviously an occasional vagrant and hence belonging to the tyrphoxenous category. More interesting is the occurrence of microcavernicolous species living in burrows of small mammals, viz. *Spelobiaczizeki* (Duda, 1918) and *S.talparum* (Richards, 1927). The mycophagous *Spelobiaparapusio* (Dahl, 1909) is interesting because only males have been found although it is a parthenogenetic species at higher latitudes (Roháček 1983). Apparently, the relatively high altitude of mire localities in the Caucasus is not an obstacle to the occurrence of bisexual populations normally occurring in southern Europe at lower altitudes.

Wing reduction and the loss of or decreased ability to fly has been observed in many different families of Diptera (e.g., Tipulidae, Limoniidae, Chironomidae, Phoridae, Chloropidae, Ephydridae, Drosophilidae, etc.), primarily in species adapted to cold and open windy habitats, and especially in those living at high latitudes and altitudes (e.g., see Hackman 1964; Byers 1969). Among Sphaeroceridae, wing reduction and/or wing loss occur most commonly among AcalyptrataeDiptera also in species living inside wet substrates (e.g., moss, litter, grass tufts, etc.) including peat-bog habitats and in the microcavernicolous species (Roháček 2012). Formerly, several brachypterous (more precisely wing-polymorphic) and even apterous species of Sphaeroceridae have been recorded from peat bogs in Europe. These include two tyrphophilous species, Phthitia (Collimosina) spinosa and *Pteremisfenestralis* (both wing-polymorphic), and several tyrphoneutral species, *Terrilimosinacorrivalis* (Villeneuve, 1918), *Pullimosinameijerei* (Duda, 1918) (both wing-polymorphic), and the apterous *Aptilotusparadoxus* Mik, 1898 (cf. Roháček 1984). Moreover, some Sphaeroceridae display a tendency to increase wing reduction at higher latitudes and altitudes, e.g., some wing-polymorphic species including *Pteremisfenestralis* (see Roháček 1975, 2012). Hence, the high-montane mire habitat of *P.turfosa* combines several features which can promote wing reduction in Sphaeroceridae.

## Supplementary Material

XML Treatment for Pullimosina (Pullimosina) turfosa

## References

[B1] Anderson R Mantell A Nelson B (2017) An invertebrate survey of Scragh Bog, Co Westmeath. Irish Wildlife Manuals, 96. National Parks and Wildlife Service, Department of Arts, Heritage, Regional, Rural and Gaeltacht Affairs, Dublin, 85 pp. https://www.npws.ie/sites/default/files/publications/pdf/IWM96.pdf

[B2] Batzer D Wu H Wheeler T Eggert S (2016) Chapter 7. Peatland invertebrates. In: Batzer D Boix D (Eds) Invertebrates in freshwater wetlands.An international perspective on their ecology. Springer, Cham – Heidelberg – New York – Dordrecht – London, 219–250. 10.1007/978-3-319-24978-0_7

[B3] Botch MS Masing VV (1979) Mire ecosystems in the USSR. Nauka, Leningrad, 188 pp. [In Russian]

[B4] Botch MS Masing VV (1983) Mire ecosystems in the U.S.S.R. In: Gore AJP (Ed.) Ecosystems of the World 4B.Mires: swamp, bog, fen and moor. Regional studies. Elsevier, Amsterdam – Oxford – New York, 95–152.

[B5] Boyce DC (2004) A review of the invertebrate assemblage of acid mires. English Nature Research Reports 592, 109 pp.

[B6] Buck M Marshall SA (2009) Revision of New World *Leptocera* Olivier (Diptera, Sphaeroceridae). Zootaxa 2039(1): 1–139. 10.11646/zootaxa.2039.1.1

[B7] Byers GW (1969) Evolution of wing reduction in crane flies (Diptera: Tipulidae). Evolution 23(2): 346–354. 10.1111/j.1558-5646.1969.tb03517.x 28562884

[B8] Carles-Tolrá M (2001) Two new *Minilimosina* Rohaček species from Andorra (Diptera, Sphaeroceridae). Boletin de la Asociacion Espanola de Entomologia 25(3–4): 9–15.

[B9] Coulson JC Butterfield JEL (1985) The invertebrate communities of peat and upland grasslands in the North of England and some conservation implications. Biological Conservation 34(3): 197–225. 10.1016/0006-3207(85)90093-X

[B10] Dahl F (1909) Die Gattung *Limosina* und die biocönotische Forschung. Sitzungsberichte der Gesellschaft naturforschender Freunde zu Berlin 6: 360–377. https://www.zobodat.at/pdf/Sitzber-Ges-Naturforsch-Freunde-Berlin_1909_0360-0377.pdf

[B11] Doskočil J (1973) Doukřídlí (Diptera, Acalyptrata) Pančiské louky v Krkonoších. Die Zweiflügler (Diptera, Acalyptrata) der Pančice-Wiese im Krkonoše Gebirge. Opera Corcontica 10: 211–224. [In Czech, with German summary]

[B12] Drake CM Godfrey A Sanderson AC (1989) A survey of the invertebrates of five lowland bogs in Cumbria. England Field Unit 60, Nature Conservancy Council, Peterborough, 113 pp. [+ Appendices 1–3]

[B13] Duda O (1938) 57. Sphaeroceridae (Cypselidae). In: Lindner E (Ed.) Die Fliegen der palaearktischen Region 6, E. Schweizerbart’sche Verlagsbuchhandlung, Stuttgart, 182 pp.

[B14] Elberg K (1969) On migrations of flies (DipteraBrachycera) on raised bogs. Eesti NSV Teaduste Akadeemia Toimetised, Bioloogia [Izvestiya Akademii nauk Estonskoy SSR] 18(3): 270–275. [In Russian, with Estonian and English summaries] 10.3176/chem.geol.1969.3.10

[B15] Elberg KYu (1971) Fauna of Acalyptrata flies (DipteraBrachycera) of mires of Estonia. Abstract of candidate of biological sciences dissertation. University of Tartu, Tartu, 24 pp. [In Russian]

[B16] Evstigneev DA Glukhova NV (2022) Tephritid flies (Diptera: Tephritidae) of the Caucasus and Transcaucasia: new records and new host plants. Zoosystematica Rossica 31(1): 118–129. 10.31610/zsr/2022.31.1.118

[B17] Evstigneev DA Przhiboro AA (2021) New records of flies of the genus *Tephritis* (Diptera: Tephritidae) from the Caucasus and Transcaucasia, with notes on other tephritid species. Zoosystematica Rossica 30(1): 13–24. 10.31610/zsr/2021.30.1.13

[B18] Florén F (1989) Distribution, phenology and habitats of the lesser dung fly species (Diptera, Sphaeroceridae) of Sweden and Norway, with notes from adjacent countries. Entomologisk Tidskrift 110: 1–29. https://www.sef.nu/download/entomologisk_tidskrift/et_1989/ET%201989%201-29w23.pdf

[B19] Haarto A Kahanpää J (2013) Notes on Finnish Sphaeroceridae (Diptera) with description of the female of *Minilimosinatenera* Rohacek, 1983. Entomologica Fennica 24(4): 228–233. 10.33338/ef.9385

[B20] Hackman W (1964) On reduction and loss of wings in Diptera. Notulae Entomologicae 44: 73–93.

[B21] Harnisch O (1925) Studien über Ökologie und Tiergeographie de Moore. Zoologische Jahrbücher. Zeitschrift für Systematik. Geographie und Biologie der Tiere, Jena 51: 1–166.

[B22] Hayashi T (2006) The genus *Pullimosina* Roháček (Diptera, Sphaeroceridae) from Japan. Japanese Journal of Sanitary Zoology 57(4): 265–272. 10.7601/mez.57.265

[B23] Holmes PR Valentine J Boyce DC Reed DK (1992) Lesser dung flies (Diptera: Sphaeroceridae) in Welsh peatlands. Dipterists Digest 8(1991): 6–13.

[B24] Joosten H Couwenberg J Moen A Tanneberger F (2017) 3. Mire and peatland terms and definitions in Europe In: Joosten H Tanneberger F Moen A (Eds) Mires and peatlands of Europe: Status, distribution and conservation.Schweizerbart Science Publishers, Stuttgart, 65–96. https://doi.org/mireseurope/2017/0001-0005

[B25] Keiper JB Walton WE Foote BA (2002) Biology and ecology of higher Diptera from freshwater wetlands. Annual Review of Entomology 47(1): 207–232. 10.1146/annurev.ento.47.091201.145159 11729074

[B26] Krogerus R (1960) Ökologische Studien über nordische Moorarthropoden. Commentationes Biologicae 21(3): 1–238.

[B27] Kuznetsova NV (1987) Fauna and ecology of Sphaeroceridae (Diptera) from maritime lowland of Latvia. Latvijas Entomologs 30: 60–70. [In Russian, with English summary]

[B28] Marshall SA (1986) A revision of the Nearctic species of the genus *Pullimosina* (Diptera, Sphaeroceridae). Canadian Journal of Zoology 64(2): 522–536. 10.1139/z86-077

[B29] Marshall SA (1994) Peatland Sphaeroceridae (Diptera) of Canada. Memoirs of the Entomological Society of Canada 169(S169): 173–179. 10.4039/entm126169173-1

[B30] Marshall SA (1997) Sphaerocerid flies (Diptera: Sphaeroceridae) of the Yukon. In: Danks HV Downes JA (Eds) Insects of the Yukon.Biological Survey of Canada (Terrestrial Arthropods), Ottawa, 663–685.

[B31] Marshall SA Finnamore AT Blades DCA (1999) 17. Canadian peatlands: Diversity and habitat specialization of the arthropod fauna. In: Batzer DP Rader RB Wissinger SA (Eds) Invertebrates in freshwater wetlands of North America.Ecology and management. John Wiley & Sons, Inc., New York – Chichester – Weinheim – Brisbane – Singapore – Toronto, 383–400.

[B32] Marshall SA Roháček J Dong H Buck M (2011) The state of Sphaeroceridae (Diptera: Acalyptratae): a world catalog update covering the years 2000–2010, with new generic synonymy, new combinations, and new distributions. Acta Entomologica Musei Nationalis Pragae 51(1): 217–298. https://www.aemnp.eu/data/article-1323/1304-51_1_217.pdf

[B33] Nelson JM (1971) The invertebrates of an area of Pennine Moorland within the Moor House Nature Reserve in northern England. Transactions of the Society for British Entomology 19(2): 173–235.

[B34] Nelson JM (1981) Some invertebrates from Blawhorn Moss National Nature Reserve, West Lothian. Forth Naturalist and Historian 6: 53–61.

[B35] Nelson JM (1982) Some invertebrates from Murder Moss (a fen near Selkirk). History of the Berwickshire Naturalist’s Club 42: 96–102.

[B36] Nelson JM Theaker JH (1982) Invertebrates on a bog within the Silver Flowe National Reserve, Galloway. Transactions of the Dumfriesshire and Galloway Natural History and Antiquarian Society, ser. 3 57: 23–28.

[B37] Pape T Beuk P Pont A Shatalkin A Ozerov A Woźnica A Merz B Bystrowski C Raper C Bergström C Kehlmaier C Clements D Greathead D Kameneva E Nartshuk E Petersen F Weber G Bächli G Geller-Grimm F Van de Weyer G Tschorsnig H de Jong H van Zuijlen J Vaňhara J Roháček J Ziegler J Majer J Hůrka K Holston K Rognes K Greve-Jensen L Munari L de Meyer M Pollet M Speight M Ebejer M Martinez M Carles-Tolrá M Földvári M Chvála M Barták M Evenhuis N Chandler P Cerretti P Meier R Rozkosny R Prescher S Gaimari S Zatwarnicki T Zeegers T Dikow T Korneyev V Richter V Michelsen V Tanasijtshuk V Mathis W Hubenov Z de Jong Y (2015) Fauna Europaea: Diptera – Brachycera. Biodiversity Data Journal 3: e4187. [31 pp.] 10.3897/BDJ.3.e4187 PMC4339814 25733962

[B38] Papp L (1973) Sphaeroceridae (Diptera) from Mongolia. Acta Zoologica Academiae Scientiarum Hungaricae 19: 369–425.

[B39] Papp L (1974) New species and records of Sphaeroceridae from Central Asia (Diptera). Annales Historico-Naturales Musei Nationalis Hungarici 66: 251–268.

[B40] Papp L (1979) New species and records of Sphaeroceridae (Diptera) from the USSR. Annales Historico-Naturales Musei Nationalis Hungarici 71: 219–230.

[B41] Papp L Roháček J (2021) 99 Sphaeroceridae (Lesser Dung Flies). In: Kirk-Spriggs AH Sinclair BJ (Eds) Manual of Afrotropical Diptera, 3.Brachycera-Cyclorrhapha, excluding Calyptratae. Suricata 8. South African National Biodiversity Institute, Pretoria, 2145–2192.

[B42] Pax F (1937) Die Moorfauna des Glatzer Schneeberges. 2 Allgemeine Charakteristik der Hochmoore. Beiträge zur Biologie des Glatzer Schneeberges. Breslau 3: 237–266.

[B43] Peus F (1928) Beiträge zur Kenntnis der Tierwelt nordwestdeutscher Hochmoore. Zeitschrift für Morphologie und Ökologie der Tiere 12: 533–683. 10.1007/BF00403122

[B44] Peus F (1932) Die Tierwelt der Moore. Handbuch der Moorkunde III. Borntraeger Verlag, Berlin, 127 pp.

[B45] Pitkin BR Coulson JC Butterfield J (1985) The distribution and biology of Sphaeroceridae (Diptera) in upland regions of northern England. Naturalist 110: 81–90.

[B46] Prokin AA Sazhnev AS Philippov DA (2019) Water beetles (Insecta: Coleoptera) of some peatlands of the North Caucasus. Nature Conservation Research 4(2): 57–66. 10.24189/ncr.2019.016

[B47] Prokina KI Philippov DA (2019) Centrohelid heliozoans (Haptista: Centroplasthelida) from mires in the North Caucasus, Russia. Mires and Peat 24, article 36: 1–20. 10.19189/MaP.2019.OMB.StA.1806

[B48] Przhiboro AA (2012) Aquatic and shore macroinvertebrates and assessment of their abundance. In: Iovchenko NP (Ed.) Ekosistemy zakaznika “Rakovye ozera”: istoriya i sovremennoe sostoyanie [Ecosystems of the nature reserve “Lakes Rakovye”: History and present state]. Trudy S.-Peterburgskogo obshchestva estestvoispytatelei [Proceedings of St. Petersburg Society of Naturalists], ser. 6, 6: 53–65, 208, 252–272 (Annexes 3 and 4) + 4 pages of photographs. St. Petersburg University Press. [In Russian with English summary]

[B49] Przhiboro A Paasivirta L (2012) Chironomidae of semiaquatic lake shore habitats in the Karelian Isthmus (northwestern Russia). Fauna Norvegica 31: 87–94. 10.5324/fn.v31i0.1410

[B50] Rabeler W (1931) Die Fauna der Göldenitzer Hochmoores in Mecklenburg. Zeitschrift für Morphologie und Ökologie der Tiere 21(1–2): 173–315. 10.1007/BF00406497

[B51] Richards OW (1930) The British species of Sphaeroceridae (Borboridae, Diptera). Proceedings of the Zoological Society of London 1930(2): 261–345. 10.1111/j.1096-3642.1930.tb00979.x

[B52] Rief S (1996) Einfluß der Bewirtschaftung auf ausgewählte Diptera (Nematocera: Limoniidae; Tipulidae; Trichoceridae; Brachycera: Empididae; Hybotidae; Dolichopodidae) verschiedener Ökosysteme auf Niedermoortorfen. Faunistisch-Ökologische Mitteilungen (Supplement 20): 47–76.

[B53] Roháček J (1975) Die Flügelpolymorphie bei den europäischen Sphaeroceridenarten und Taxonomie der *Limosinaheteroneura*-Gruppe (Diptera). Acta Entomologica Bohemoslovaca 72: 196–207.

[B54] Roháček J (1982a) Revision of the subgenus Leptocera (s. str.) of Europe (Diptera, Sphaeroceridae). Entomologische Abhandlungen. Staatliches Museum für Tierkunde in Dresden 46(1): 1–44.

[B55] Roháček J (1982b) A monograph and re-classification of the previous genus *Limosina* Macquart (Diptera, Sphaeroceridae) of Europe. Part I. Beiträge zur Entomologie, Berlin 32: 195–282. https://www.contributions-to-entomology.org/article/view/1178/1177

[B56] Roháček J (1983) A monograph and re-classification of the previous genus *Limosina* Macquart (Diptera, Sphaeroceridae) of Europe. Part II. Beiträge zur Entomologie, Berlin 33: 3–195. https://www.contributions-to-entomology.org/article/view/1184/1183

[B57] Roháček J (1984) Acalypterate Diptera of peat bogs in North Moravia (Czechoslovakia). Part 6. Sphaeroceridae. Časopis Slezského Muzea, Opava (A) 33: 97–131.

[B58] Roháček J (1985) A monograph and re-classification of the previous genus *Limosina* Macquart (Diptera, Sphaeroceridae) of Europe. Part IV. Beiträge zur Entomologie, Berlin 35: 101–179. https://www.contributions-to-entomology.org/article/view/1209/1208

[B59] Roháček J (1990) New species of Limosininae (Diptera, Sphaeroceridae) from northern Europe. Acta Entomologica Bohemoslovaca 87: 221–231.

[B60] Roháček J (1991) A monograph of *Leptocera* (*Rachispoda* Lioy) of the West Palaearctic area (Diptera, Sphaeroceridae). Časopis Slezského zemského Muzea, Opava (A) 40: 97–288.

[B61] Roháček J (1998) 3.43. Family Sphaeroceridae. In: Papp L Darvas B (Eds) Contributions to a Manual of Palaearctic Diptera, 3.Higher Brachycera. Science Herald, Budapest, 463–496.

[B62] Roháček J (2001) The type material of Sphaeroceridae described by J. Villeneuve with lectotype designations and nomenclatural and taxonomic notes (Diptera). Bulletin de la Société entomologique de France 105(5) (2000): 467–478. 10.3406/bsef.2000.16706

[B63] Roháček J (2010) West Palaearctic Minilimosina (Svarciella): a new species, new records, key and taxonomical notes (Diptera: Sphaeroceridae). Časopis Slezského Zemského Muzea Opava (A), 58(2009): 97–114.

[B64] Roháček J (2012) Wing polymorphism in European species of Sphaeroceridae (Diptera). Acta Entomologica Musei Nationalis Pragae 52(2): 535–558. https://www.aemnp.eu/data/article-1389/1370-52_2_535.pdf

[B65] Roháček J (2019) First Sphaeroceridae (Diptera) endemic to Madeira – three new terricolous species of *Spelobia* and *Pullimosina*. Acta Entomologica Musei Nationalis Pragae 59(1): 107–124. 10.2478/aemnp-2019-0009

[B66] Roháček J Barták M (1999) Sphaeroceridae (Diptera) of peat-bogs in the Šumava Mts. (SW Bohemia, Czech Republic). Časopis Slezského zemského muzea Opava (A) 48: 9–32.

[B67] Roháček J Máca J (1982) Acalypterate Diptera of peat-bogs in North Moravia (Czechoslovakia). Part 2. Ecological classification, Opomyzidae, Anthomyzidae, Asteiidae, Diastatidae, Drosophilidae. Časopis Slezského Muzea, Opava (A) 31: 193–213.

[B68] Roháček J Marshall SA (1988) A review of Minilimosina (Svarciella) Rohácek, with descriptions of fourteen new species (Diptera: Sphaeroceridae). Insecta Mundi 2: 241–282.

[B69] Roháček J Przhiboro AA (2022) Anthomyzidae (Diptera) in peat bogs of the North Caucasus (Russia). Entomological Review 101(8)(2021): 1188–1194. 10.1134/S0013873821080157

[B70] Roháček J Marshall SA Norrbom AL Buck M Quiros DI Smith I (2001) World catalog of Sphaeroceridae (Diptera). Slezské zemské muzeum, Opava, 414 pp.

[B71] Roháček J Kubík Š Barták M (2005) Sphaeroceridae. In: Barták M Kubík Š (Eds) Diptera of Podyjí National Park and its environs.Česká zemědělská univerzita v Praze, Praha, 335–348.

[B72] Salmela J (2004) Semiaquatic flies (Diptera, Nematocera) of three mires in the southern boreal zone, Finland. Memoranda Societatis Pro Fauna et Flora Fennica 80: 1–10.

[B73] Spitzer K Danks HV (2006) Insect biodiversity of Boreal peat bogs. Annual Review of Entomology 51(1): 137–161. 10.1146/annurev.ento.51.110104.151036 16332207

[B74] Spungis V (2008) Fauna and ecology of terrestrial invertebrates in raised bogs in Latvia. Latvijas Entomologs (Supplementum 6): 1–84.

[B75] Stuke J-H Roháček J (2019) Die Kleinen Dungfliegen Niedersachsens und Bremens (Diptera: Sphaeroceridae). Entomologische Zeitschrift Schwanfeld 129(1): 19–47.

[B76] Su L (2011) Lesser Dung Flies. Liaoning University Press, Shenyang, Liaoning, China, 229 pp. [In Chinese]

[B77] Su L-X Liu G-C Xu J (2013) Genus *Pullimosina* (Diptera: Sphaeroceridae) in China with description of a new species. Entomologica Fennica 24(1): 1–8. 10.33338/ef.84594

[B78] Sushko G (2012) The insect fauna of «Yelnia» peat bog, north-west Belarus. LAP Lambert Academic Publishing, Saarbrücken, 104 pp.

[B79] Taillefer AG Wheeler TA (2010) Effect of drainage ditches on Brachycera (Diptera) diversity in a Southern Quebec peatland. Canadian Entomologist 142(2): 160–172. 10.4039/n09-062

[B80] Taillefer AG Wheeler TA (2011) Community assembly of Diptera following restoration of mined boreal bogs: Taxonomic and functional diversity. Journal of Insect Conservation 16(2): 165–176. 10.1007/s10841-011-9403-x

[B81] Vitt DH (1994) An overview of factors that influence the development of Canadian peatlands. Memoirs of the Entomological Society of Canada 169(S169): 7–20. 10.4039/entm126169007-1

[B82] Zatwarnicki T (1996) A new reconstruction of the origin of eremoneuran hypopygium and its implications for classification (Insecta: Diptera). Genus 7: 103–175.

